# Consumer Perceptions and Behaviors Toward Fermented Foods: Insights From a Canadian Survey

**DOI:** 10.1002/fsn3.72287

**Published:** 2026-08-28

**Authors:** Camila Schultz Marcolla, Samantha Tapping, Carla Liria Sánchez‐Lafuente, Kara Sampsell, Gabrielle Eaton, Ellen Goddard, Benjamin P. Willing, Jeremy P. Burton, Raylene A. Reimer

**Affiliations:** ^1^ Department of Agricultural, Food and Nutritional Science University of Alberta Edmonton Alberta Canada; ^2^ Department of Microbiology and Immunology Western University London Ontario Canada; ^3^ Canadian Fermented Foods Initiative London Ontario Canada; ^4^ Faculty of Kinesiology University of Calgary Calgary Alberta Canada; ^5^ Department of Resource Economics and Environmental Sociology University of Alberta Alberta Canada; ^6^ Lawson Research Institute London Ontario Canada; ^7^ Division of Urology, Department of Surgery Western University London Ontario Canada; ^8^ Department of Biochemistry and Molecular Biology University of Calgary Calgary Alberta Canada

**Keywords:** Canadian survey, consumer attitudes, consumer patterns, fermented foods, knowledge gaps, perceptions

## Abstract

Chronic and autoimmune diseases continue to rise across Canada, demonstrating the need for sustainable dietary strategies that promote long‐term health. Fermented foods (FFs) have emerged as a potential approach, yet national data on consumption patterns and public perceptions remain largely unknown. A cross‐sectional survey of 4045 Canadian adults was conducted (September–November 2023) to assess consumption patterns, including behaviors, perceptions, and general knowledge about FFs and fermentation practices. Overall, 25.7% of participants reported consuming all 18 surveyed FFs at least once. Cheese (95.7%) and yogurt (93.2%) were the most consumed, while fermented fish (38.9%) and fermented cereals/grains (40.2%) were least consumed. Consumption patterns varied by age, sex, income, and education. Lower intake was seen in older adults, those with lower income and education, and those for whom health was not a primary consideration in food choice. Taste was the main motivator for consumption, with health second. Home fermentation was uncommon (9.0%) but more frequent among younger, more educated individuals. While there was a willingness to try new products (29.1%) and engage in home fermentation (30.0%), participants expressed uncertainty about FF safety (18.4%) and potential health benefits (52.7%). Nonetheless, two‐thirds wanted to learn more about FFs, and nearly half preferred guidance from trusted sources such as Canada's Food Guide. This study is the first comprehensive assessment of FF consumption patterns and perceptions across Canada, revealing sociodemographic trends, knowledge gaps, and opportunities to strengthen consumer education, evidence‐based resources, and future public health initiatives related to FFs.

## Graphical abstract

Canadian Survey of Consumer Perceptions and Behaviors Toward Fermented Foods. Overview of the study design, key findings, and implications of a nationally representative online survey of 4045 Canadian adults examining fermented food consumption, behaviors, perceptions, and knowledge. Results highlight common drivers and barriers to consumption, knowledge and safety‐related uncertainties, sociodemographic influences on fermented food intake, and opportunities for targeted education, consumer engagement, and trusted public health resources.

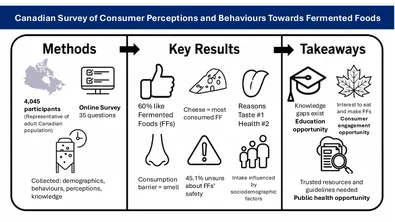

## 1 Introduction

Noncommunicable diseases, including cancers, cardiovascular diseases, metabolic, neurodegenerative, and autoimmune disorders are rising in Western populations (Adolph and Tilg 2024; Watson et al. 2025). In Canada, data from 2021 showed that almost 45% of people aged 12 and older were living with at least one chronic condition, and about 8% were managing three or more. These figures are expected to be even higher today (Statistics Canada 2023). This public health burden parallels changes to dietary patterns characterized by reduced fruit and vegetable intake and increasing reliance on highly processed convenience foods high in calories, saturated fat, sugar, and sodium (Adolph and Tilg 2024; Hall et al. 2019) that are associated with all‐cause mortality (Taneri et al. 2022). Despite sustained public health efforts, population‐level adherence to recommended dietary patterns remains limited with barriers rooted in cost, time, taste, perceived benefit, and insufficient nutrition education (Domosławska‐Żylińska et al. 2024; Leme et al. 2021). Simultaneously, global food systems are facing increasing pressures from climate change, land degradation, and a growing population (Graham and Ledesma‐Amaro 2023). Consequently, there is an urgent need for dietary approaches that are nutritionally beneficial, culturally acceptable, environmentally sustainable, and supported by effective education that empowers individuals to make informed choices.

Fermented foods (FFs) represent one possible opportunity. Although fermentation has been practiced for centuries as a method of preservation and spoilage prevention, there has been a recent resurgence of interest in its ability to create distinct sensory properties. By consensus, FFs are defined as “foods made through the desired microbial growth and enzymatic conversions of food components” (Marco et al. 2021). These microbial processes generate an array of bioactive metabolites, including peptides, short‐chain fatty acids (SCFAs), and vitamins (Caffrey et al. 2025; Casertano et al. 2022). Studies show that these molecules can enhance nutrient bioavailability, improve food safety, and act as signaling agents that interface with the gastrointestinal microbiota, metabolic networks, and the immune system (Shah et al. 2023). FFs may also introduce microorganisms that provide benefits to the host even when they are present only transiently or in nonviable forms (Marco et al. 2017). Growing evidence from human trials and mechanistic studies continues to support these effects and their role in preventing or helping to ameliorate various chronic diseases [reviewed in (Sampsell et al. 2025)].

Fermented foods have long held a place in global diets, yet evidence from several regions suggests a decline in home fermentation practices alongside limited public knowledge about these foods (Tamang et al. 2020). Surveys from Slovenia show that younger generations have minimal hands‐on experience with traditional fermentation and only a partial understanding of fermentation processes and their associated health effects (Šikić‐Pogačar et al. 2022). Similarly, national dietary surveillance data from Korea demonstrate a long‐term decrease in the consumption of FFs, even within a culture historically rooted in fermentation practices (Kim et al. 2020).

While fermentation has also been an integral part of Canada's culinary tradition, its contemporary role in Canadian diets and the extent of current knowledge and practices remain unclear. Given the cultural importance, sustainability implications, and potential health benefits associated with FFs, understanding how Canadians perceive, consume, and value these foods is essential. Such insights can inform researchers, industry stakeholders and policy makers, helping to shape future research priorities, guide educational initiatives, and support their integration into national dietary recommendations. To gain this understanding, a cross‐sectional survey of 4045 Canadian adults was conducted to examine consumption patterns, knowledge, and perceptions of FFs. The study aimed to address several key questions: which FFs are most and least commonly consumed in Canada; which sociodemographic and lifestyle factors influence the number of FF products consumed; what are the primary drivers and barriers to consumption; and what emerging trends and opportunities exist in this area. By integrating insights on consumption patterns with behavioral factors, this study provides a comprehensive national snapshot of how Canadians engage with FFs.

## 2 Methods

### 2.1 Study Design, Ethics and Participant Details

This cross‐sectional study recruited participants through Qualtrics Research Services and institutional communication channels at the Canadian research institutions of Western University, Lawson Research Institute, University of Alberta and University of Calgary. Ethics approval was granted by the Health Sciences Research Ethics Board at Western University (HSREB #123285), by Lawson Research Institute (Project ID: 123285, ReDA 13064, Review Reference: 2023‐123285‐83318), and the Conjoint Health Ethics Research Board at University of Alberta and University of Calgary (REB23‐0897). The survey was open from September to October 2023. Qualtrics Research Services Online Sample service contacted individuals who had a pre‐existing agreement with the Qualtrics Company to be contacted regarding surveys they may choose to complete. Quotas were utilized to reach a target sample (*n* = 4000) that reflected the demographic spread of Canadians in terms of age, sex, rural vs. urban residency, and race/ethnicity. The remaining participants were recruited locally via institutional networks. Prior to initiating the questionnaire, participants were provided with background information on the study's objectives and were required to provide informed consent to complete a one‐time survey. Eligibility criteria included adults aged 18 years or older residing in Canada.

### 2.2 Data Collection

The survey consisted of 35 questions composed of multiple‐choice, checkbox lists, and free text responses (Table S1). The survey was administered using Qualtrics XM (Qualtrics, Provo, UT, USA), configured to reflect Canadian demographics, and included questions on sociodemographic characteristics as well as perceptions and behaviors related to FFs. The questionnaire was developed and reviewed by the research team for clarity and content relevance based on availability of FF products in Canada and current literature on FF consumption and perceptions. Prior to full deployment, the survey underwent review by Qualtrics Research Services to verify survey setup, target audience specifications, screener logic, and survey flow. The survey was initially launched as a soft launch (~10% of the target sample), allowing review of incoming data and identification of any operational issues before full field deployment. Formal cognitive interviewing and psychometric validation procedures were not conducted, as responses were mainly analyzed individually and not part of a composite measure. Responses were subject to quality‐control procedures including speed checks and duplicate detection.

The first survey question screened eligibility by age, automatically ending the survey for participants under 18. The subsequent questions collected information on participants' province/territory of residence, weight, height, education level, marital status, household composition, number of children, ethnicity/race, generational status in Canada, area of residency, self‐perceived health status, frequency of family doctor visits, consideration of health benefits when choosing foods, and dietary patterns. The second section of the survey examined behaviors surrounding FFs. Participants indicated whether they consumed each of the listed 18 products, and for those they consumed, reported quantity of intake using predefined categories such as: “1 time per month or less,” “2–3 times per month,” “1 time per day,” “2–3 times per day,” “4+ times per day,” etc. Later questions assessed primary motivations for consumption of each listed FF, including options such as “the product tastes good”, “promotes overall health”, “to manage a disease/condition”, “cultural/heritage tradition” or “other”. Other questions related to behavior included the consumption of non‐heated FF products, whether children in their household consumed FFs, engagement in home fermentation, and resources used for home fermentation. The third section addressed perceptions and knowledge. Participants were asked about willingness to prepare FFs at home, perceived availability of FFs, cultural and generational traditions, taste, and health perceptions. Knowledge‐related questions assessed participants' understanding of the differences between probiotics and FFs, the role of microorganisms in fermented foods, perceptions around FF safety, and interest in educational resources. See the full survey in Supplementary Tables S1 and S2.

### 2.3 Statistical Analyses

All answers were tabulated, including the means and standard deviations of numerical variables and the percentage of responses of categorical variables. Differences between continuous variables were analyzed using one‐way ANOVA for means and Kruskal‐Wallis tests for medians. Categorical variables were analyzed using the chi‐squared method. An ordinary least squares regression model was used to identify factors associated with the total number of FFs consumed and the frequency of consumption. To this end, the ranked frequencies of consumption of individual products and the total number of products consumed reported by each participant were summed into a variable named “consumption score”. The “consumption score” was developed as a mean to access the overall engagement of participants with FFs by incorporating the diversity and frequency of products consumed into a single measurement, rather than actual quantities consumed of each individual product. Consumption score could range from 0 (for individuals who didn't consume any of the listed products) to 180 (for individuals reporting consuming all the listed products, 3 to 4 times per day). All food products contributed equally for this composite measure. Demographic factors, health and health perceptions, behaviors and perceptions on FFs, and family and cultural history factors were included in the model as independent variables (for a total of 28 variables). Answers that were not provided (“unknown” or “missing”) were combined to the most frequent answers for qualitative variables or transformed into the mean of the dataset for quantitative variables (Supplementary Methods file). Multicollinearity testing was performed by calculating variance inflation factor and tolerance values using *car* package in R (Fox and Weisberg 2019). Correlations between explanatory variables were calculated using stats package in R and visualized using *corrplot* package in R. Effect plots were created to show the predicted values of the outcome variable for different levels of each explanatory variable using *effects* package in R (Fox and Weisberg 2019). Consumption patterns of individual products were also evaluated by comparing the responses of people who consume or do not consume the products. To this end, categorical variables were analyzed using chi‐square test with Bonferroni correction and continuous variables (age and BMI) were compared across groups using the Kruskal–Wallis test. The category for “Unknown” was excluded from the chi‐square analyses.

## 3 Results

### 3.1 Participant Demographics

Table 1 summarizes participant demographics. Among 4045 Canadian participants (mean age, 47.5 ± 17.5 years), 51.7% were female, 72.6% were white, 44.1% resided in Ontario, and half reported a family residence in Canada for three or more generations. The largest percentage (25.7%) reported an annual income of $51,000–80,000, and nearly one third (31.4%) held an undergraduate degree. Fewer than half were married (43.5%), most reported no children (66.7%), and 42.5% resided in urban areas with two to four household members (68.3%). The mean BMI was in the overweight range (27.1%), and three quarters self‐identified as healthy. Most participants followed an omnivorous diet (89.1%), and the majority visited a physician two to four times per year. When selecting food items, 44% reported considering health effects “most of the time.” Nearly half reported no cultural history of FF consumption.

**TABLE 1:** Respondent characteristics (*n* = 4045).

Variable	*N* or average value	(%)
Age, years		
Mean (standard deviation)	47.5 (17.5)	
Median (IQR)	47.0 (33.0–62.0)	
18–30	833	20.6
31–40	746	18.4
41–50	679	16.8
51–60	654	16.2
61–70	727	18
70–80	351	8.7
> 81	55	1.4
Sex		
Female	2091	51.7
Male	1933	47.8
Other	18	0.4
Unknown	3	0.1
BMI, kg/m^2^		
Mean (standard deviation)	27.1 (5.0)	
Median (IQR)	26.2 (22.8–30.7)	
Ethnicity		
Indigenous	99	2.4
Asian	722	17.8
Black	206	5.1
White	2936	72.6
Hispanic or Latina	70	1.7
Other/Not listed	5	0.1
Prefer not to share	7	0.2
Income, $CAD		
< 30,000	684	16.9
30,000‐50,000	801	19.8
51,000‐80,000	1024	25.3
81,000‐120,000	839	20.7
> 120,000	672	16.6
Unknown	25	0.6
Highest level of education		
Community college	882	21.8
Elementary to high school	286	7.1
Trade school	1004	24.8
University (professional/post‐graduate/doctoral)	1270	31.4
University (undergraduate)	599	14.8
Unknown	4	0.1
Geographic Location		
Alberta	504	12.5
British Columbia	534	13.2
Manitoba	191	4.7
New Brunswick	104	2.6
Newfoundland and Labrador	92	2.3
Nova Scotia	129	3.2
Ontario	1783	44.1
Prince Edward Island	29	0.7
Quebec	489	12.1
Saskatchewan	127	3.1
Northwest Territories	1	0.0
Nunavut	1	0.0
Yukon	3	0.1
Canadian Outside Canada	58	1.4
Living Area		
Rural	809	20.0
Suburban	1518	37.5
Urban	1718	42.5
Number of household members		
1 (I live alone)	922	22.8
2–4	2764	68.3
More than 4	357	8.8
Unknown	2	0.0
Number of children		
None	2699	66.7
1	674	16.7
2–4	654	16.2
More than 4	13	0.3
Unknown	5	0.1
Dietary pattern		
Omnivorous	3606	89.1
Pescatarian	207	5.1
Vegan	41	1.0
Vegetarian	175	4.3
Unknown	16	0.4

### 3.2 Observed Behaviors Around Fermented Foods

**FIGURE 1:**
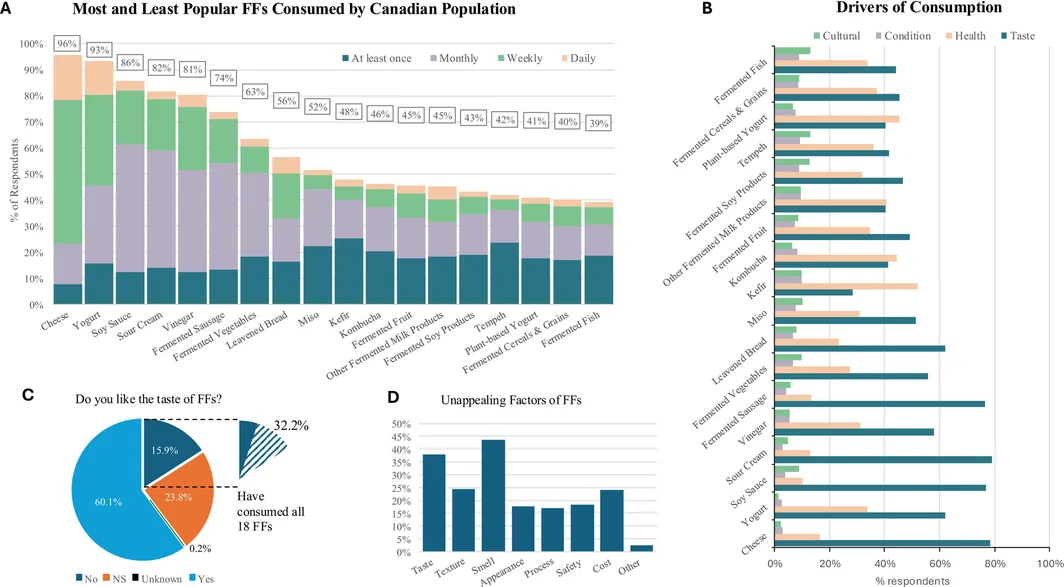
Consumption patterns of surveyed fermented foods. (A) Distribution of fermented foods (FFs) consumed by participants, including frequency of consumption for each item. (B) Reasons for FFs consumption. Stacked bar graphs show the distribution of reasons for consuming each surveyed FFs. Across all items, taste was the most frequently cited reason, followed by health benefits. (C) Responses to whether participants liked the taste of FFs. The majority reported liking the taste; among those who did not, 32.2% had nonetheless tried all 18 surveyed FFs at least once. (D) Reported unappealing factors of FFs. Taste was the most mentioned deterrent, followed by smell. Percentages based on total sample (*N* = 4045).

A quarter of participants (25.7%) reported consuming all 18 of the FFs, whereas approximately one in a hundred consumed none. On average, participants consumed 10.7 ± 5.8 FF (Figure 1). Cheese and yogurt were consumed by nearly all participants (95.7% and 93.4%), while soy sauce (85.7%), sour cream (81.8%), and vinegar (80.5%) were also frequently reported. Plant‐based yogurt (41.1%), fermented cereals/grains (40.4%), and fermented fish (39.2%) were consumed the least (Figure 1A). Consumption patterns varied by food type: cheese, yogurt, and leavened bread were typically consumed weekly or daily, vinegar was most often consumed weekly, while soy sauce, sour cream, and fermented sausage were reported on a monthly basis. Kefir was tried by over half of participants (53%), though only 6% of those who consumed it did so on a daily basis.

Taste was the most common reason for consuming FFs, followed by health (Figure 1B). Cultural reasons accounted for a small proportion of responses and were associated with less commonly consumed foods such as fermented fish (Figure 1B). Fermented vegetables, kombucha, kefir, and plant‐based yogurt were most often consumed due to their perceived health benefits (Figure 1B). Open‐ended responses regarding the perceived healthfulness of fermented foods most referred to gut health (*n* = 488), microorganisms or probiotics (*n* = 300), and diet or nutritional benefits (*n* = 125) (Table S10). Most participants reported liking FFs (60.1%), while 15.9% did not (Figure 1C). Interestingly, of the 15.9% of participants who did not like the taste of FFs, 32.2% of them had tried all 18 of the FFs from the survey list (Figure 1C). Among unappealing attributes, smell (43.7%) and taste (38.0%) were most common, while cost (24.2%), safety concerns (18.4%), and processing (17.0%) were chosen less among participants (Figure 1D).

### 3.3 Perceptions Around Fermented Foods

**FIGURE 2:**
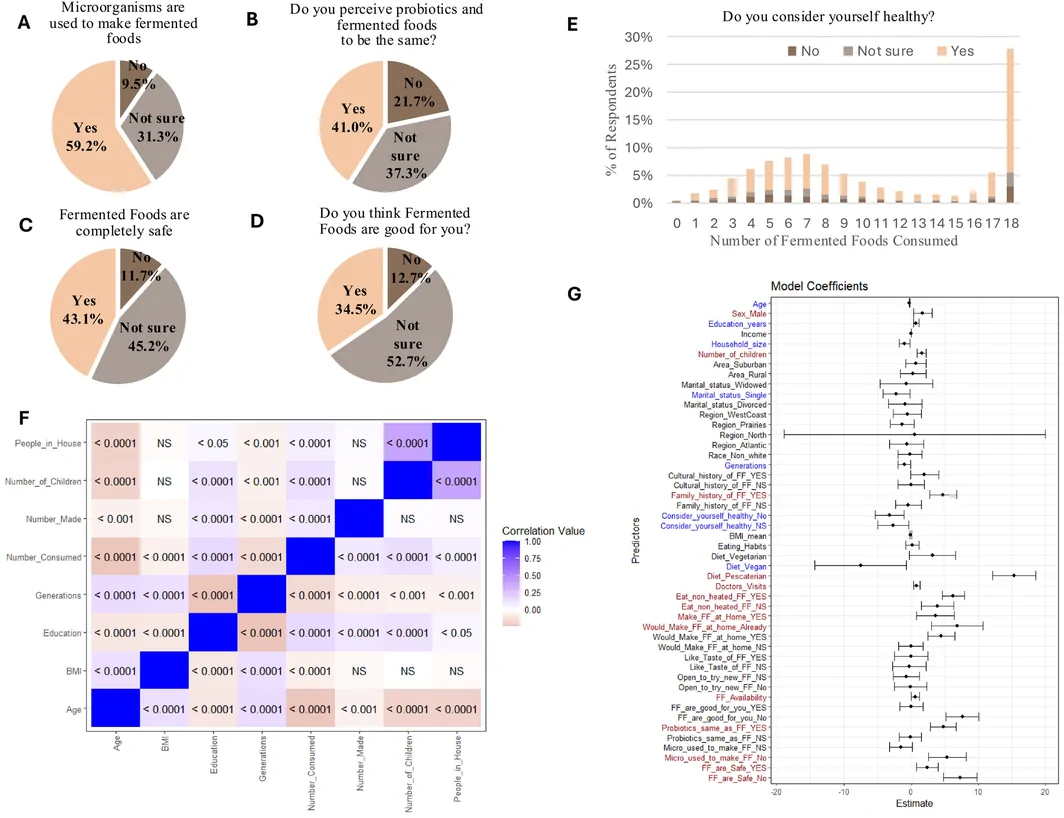
Consumer perceptions, understanding, and determinants of fermented food consumption. (A–D) Pie charts illustrating respondents' understanding of what constitutes FFs, as well as their perceptions of FF safety and health benefits. (E) Correlation matrix with the number of FFs consumed and made at home and demographic factors. Blue and purple show (F) relationship between self‐reported health consciousness and the number of different FFs consumed. The majority of health‐conscious respondents reported consuming all surveyed FFs. (G) Predictors of FF consumption scores identified through regression analysis, highlighting key factors positively (red) and negatively (blue) associated with higher intake.

Most participants recognized that microorganisms are used in fermentation, while approximately 1 in 3 (31.3%) reported uncertainty (Figure 2A). Knowledge about this was highest among those aged 66 years and older, and lowest among those aged 18–25 (Table S2). When asked if FF and probiotics were the same, 21.7% indicated they were different, 41.0% responded that they were the same, and 37.3% were unsure (Figure 2B; Table S3). Uncertainty regarding safety was common, with 45.2% of participants unsure and the highest proportion of “unsafe” responses observed in those 18–25 years old (Figure 2C; Table S4). Overall, more than half of participants were unsure if FFs were “good for them” and 1 in 3 believed they were (Figure 2D). Among participants, 72.4% of those consuming all 18 FFs considered themselves healthy (Figure 2E).

### 3.4 Factors Affecting Perceptions and Behaviors Toward Fermented Foods

Correlation analyses were performed to examine relationships between demographic characteristics and FF perceptions and behaviors. Age (*r* = −0.241, *p* < 0.0001), BMI (*r* = −0.062, *p* < 0.0001), and the number of generations in Canada (*r* = −0.152, *p* < 0.0001) were negatively associated with a higher number of FF consumed, while education level (*r* = 0.160, *p* < 0.0001), income level (*r* = 0.079, *p* < 0.0001), household size (*r* = 0.071, *p* < 0.0001), and number of children (*r* = 0.145, *p* < 0.0001) were positively associated with FF consumption (Figure 2F; Table 2; Table S9). There was also a negative association between age (*r* = −0.055, *p* < 0.0001), BMI (*r* = −0.033, *p* = 0.06), and the number of generations in Canada (*r* = −0.074, *p* < 0.0001) with the number of FFs made at home. In contrast, education level (*r* = 0.101, *p* < 0.0001) and income level (*r* = 0.038, *p* = 0.015) showed a positive association with homemade FF preparation (Figure 2F; Table S5).

**TABLE 2:** Number of fermented foods consumed by respondents.

Characteristics	How many fermented foods do you eat? *N* (% based on *N* = 4045)				
	1–5	6–10	11–15	16–18	χ^2^ (*p*)
Totals	903 (22.3)	1337 (33.1)	378 (9.3)	1409 (34.8)	
Age					
Mean (standard deviation)	50.7 (18.1)	51.9 (17.0)	46.0 (15.9)	41.7 (15.6)	(< 0.0001*)
Median (IQR)	53 (35–66)	54 (38–66)	45.5 (33–60)	39 (29–53)	277.04 (< 0.0001*)
18–25	111 (12.3)	105 (7.9)	42 (11.1)	257 (18.2)	300.04 (< 0.0001*)
26–35	120 (13.3)	184 (13.8)	72 (19.0)	323 (22.9)	
36–45	122 (13.5)	200 (15.0)	75 (19.8)	285 (20.2)	
46–55	130 (14.4)	224 (16.8)	74 (19.6)	243 (17.2)	
56–65	191 (21.2)	271 (20.3)	63 (16.7)	172 (12.2)	
66+	229 (25.4)	353 (26.4)	52 (13.8)	129 (9.2)	
BMI					
Mean (standard deviation)	27.1 (5.4)	27.7 (5.5)	27.5 (5.6)	26.4 (5.2)	(< 0.0001*)
Median (IQR)	25.9 (24–30)	27.0 (24–31)	26.6 (23–31)	25.6 (24–30)	33.545 (< 0.0001*)
Canadian Province					87.45 (< 0.0001**)
Alberta	93 (10.3)	189 (14.1)	48 (12.7)	173 (12.3)	
British Columbia	100 (11.1)	214 (16.0)	59 (15.6)	157 (11.1)	
Manitoba	46 (5.1)	68 (5.1)	17 (4.5)	60 (4.3)	
Newfoundland and Labrador	26 (2.9)	39 (2.9)	6 (1.6)	20 (1.4)	
New Brunswick	28 (3.1)	34 (2.5)	4 (1.1)	37 (2.6)	
Nova Scotia	39 (4.3)	41 (3.1)	6 (1.6)	43 (3.1)	
Northwest Territories	0 (0.0)	0 (0.0)	0 (0.0)	1 (0.1)	
Nunavut	1 (0.1)	0 (0.0)	0 (0.0)	0 (0.0)	
Ontario	407 (45.1)	545 (40.8)	177 (46.8)	646 (45.8)	
Prince Edward Island	9 (1.0)	9 (0.7)	3 (0.3)	8 (0.6)	
Quebec	122 (13.5)	125 (9.3)	45 (11.9)	195 (13.8)	
Saskatchewan	22 (2.4)	56 (4.2)	4 (1.1)	45 (3.2)	
Yukon	0 (0.0)	1 (0.1)	1 (0.3)	1 (0.1)	
Outside of Canada	10 (1.1)	16 (1.2)	8 (2.1)	23 (1.6)	
Sex					46.654 (< 0.0001**)
Male	435 (48.2)	561 (42.0)	163 (43.1)	763 (54.3)	
Female	462 (51.2)	770 (57.6)	213 (56.3)	639 (45.4)	
Other	6 (0.7)	6 (0.4)	2 (0.5)	4 (0.3)	
Highest Level of Education					134.64 (< 0.0001*)
Elementary	284 (31.5)	296 (22.1)	52 (13.8)	243 (17.2)	
Trade School	53 (5.9)	94 (7.0)	23 (6.1)	115 (8.2)	
Community College	242 (26.8)	366 (27.4)	89 (23.5)	303 (21.5)	
University (undergrad)	222 (24.6)	418 (31.3)	146 (38.6)	482 (34.2)	
University (grad)	102 (11.3)	163 (12.2)	68 (18.0)	266 (18.9)	
Income Level					92.936 (< 0.0001*)
< 30,000	209 (23.1)	176 (13.2)	53 (14.0)	246 (17.5)	
30,000–50,000	233 (25.8)	253 (18.9)	59 (15.6)	256 (18.2)	
51,000–80,000	192 (21.3)	379 (28.3)	97 (25.7)	363 (25.8)	
81,000–120,000	165 (18.3)	281 (21.0)	89 (23.5)	304 (21.6)	
> 120,000	104 (11.5)	248 (18.5)	80 (21.2)	240 (17.0)	
Living Area					10.133 (0.119)
Rural	186 (20.6)	297 (22.2)	61 (16.1)	262 (18.6)	
Suburban	328 (36.3)	495 (37.0)	149 (39.4)	537 (38.1)	
Urban	389 (43.1)	545 (40.8)	168 (44.4)	610 (43.3)	
Consider yourself healthy?					39.283 (< 0.0001*)
Not Sure	106 (11.7)	170 (12.7)	35 (9.3)	117 (8.3)	
No	159 (17.6)	182 (13.6)	53 (14.0)	157 (11.1)	
Yes	638 (70.7)	985 (73.7)	290 (76.7)	1134 (80.5)	
How often go to the doctor?					23.824 (0.005*)
0	165 (18.3)	177 (13.2)	59 (15.6)	180 (12.8)	
1	315 (34.9)	432 (32.3)	122 (32.3)	489 (34.7)	
2–4	329 (36.4)	586 (43.8)	156 (41.3)	602 (42.7)	
5 or more	94 (10.4)	142 (10.6)	41 (10.8)	138 (9.8)	
Consider health benefits when you choose food to eat?					65.249 (< 0.0001*)
Never	66 (7.3)	47 (3.5)	10 (2.6)	56 (4.0)	
Sometimes	454 (50.3)	595 (44.5)	139 (36.8)	625 (44.4)	
Most of the time	317 (35.1)	581 (43.5)	187 (49.5)	562 (39.9)	
Always	66 (7.3)	114 (8.5)	42 (11.1)	166 (11.8)	
Dietary patterns					96.935 (< 0.0001**)
Omnivorous	820 (91.3)	1260 (94.3)	331 (87.6)	1181 (83.8)	
Pescatarian	26 (2.9)	32 (2.4)	26 (6.9)	122 (8.7)	
Vegetarian	40 (4.5)	32 (2.4)	16 (4.2)	85 (6.0)	
Vegan	12 (1.3)	12 (0.9)	2 (0.5)	14 (1.0)	

* Indicates statistically significant differences between groups.

** Chi‐squared approximation unreliable due to low frequencies.

To further explore these associations and to account for consumption frequency rather than just product count, an ordinary least squares regression model was conducted with a newly created variable called the consumption score as the outcome. This score was calculated by summing both the frequency of consumption for each fermented product and the total number of different products consumed by each participant. The analysis showed that older age was associated with lower FF consumption (β = −0.179, 95% CI [−0.223, −0.134], *p* < 0.0001), while higher education years were positively associated (β = 0.628, 95% CI [0.303, 0.953], *p* < 0.0001). Individuals who were single (β = −2.230, 95% CI [−3.881, −0.579], *p* = 0.008) or from larger households (β = −0.844, 95% CI [−1.501, −0.188], *p* = 0.012) reported consuming fewer FFs. Conversely, having more children was positively correlated to FF consumption (β = 1.440, 95% CI [0.852, 2.028], *p* < 0.0001) (Figure 2G). Dietary preferences were also influential: being pescatarian was strongly associated with higher FF intake (β = 13.7, 95% CI [10.76, 16.64], *p* < 0.0001), while being vegan was associated with lower intake (β = −10.4, 95% CI [−16.65, −4.15], *p* = 0.0001). Finally, a higher number of doctor visits was linked to greater FF consumption (β = 0.578, 95% CI [0.182, 0.974], *p* = 0.004) (Figure 2G).

### 3.5 Fermented Food Availability and Home Fermentation

**FIGURE 3:**
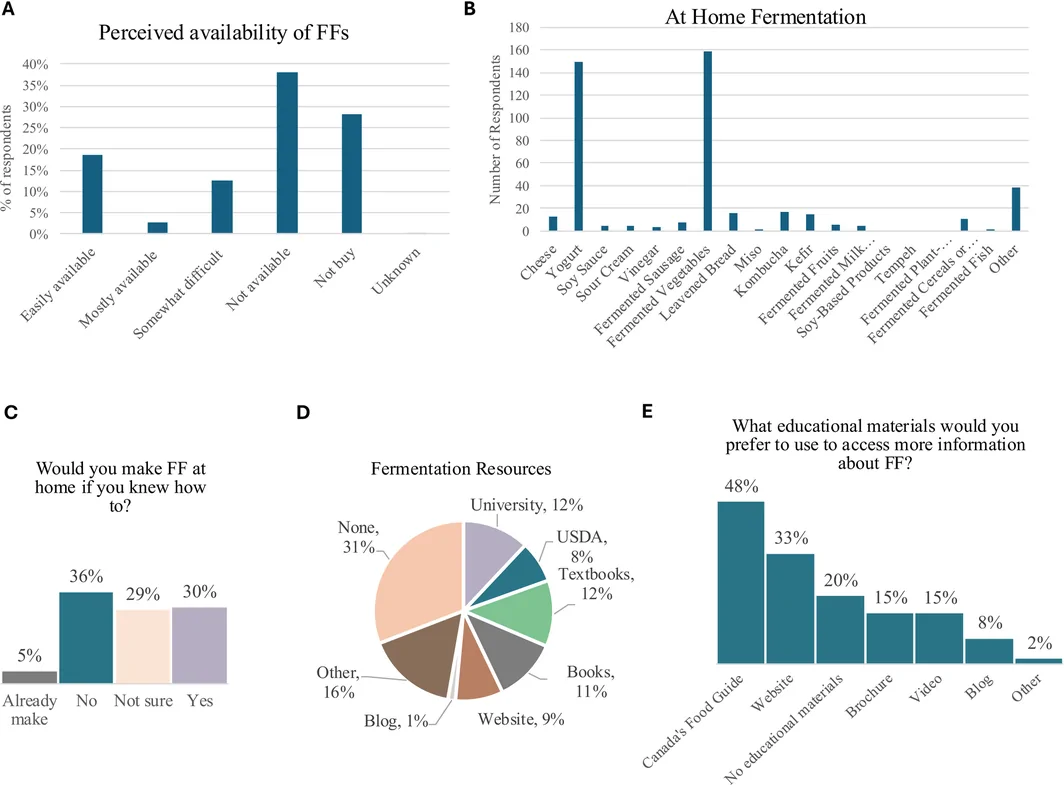
Accessibility and at‐home fermentation practices. (A) Responses to whether consumers found the FFs they wanted easy to find in stores; the majority responded “no.” (B) Number of respondents engaging in home fermentation for each surveyed product (based on the 5% who reported making FFs at home). (C) Participant interest in learning how to make FFs at home, indicating a general predisposition to do so. (D) Most commonly used resources for home fermentation. (E) Most desired sources of educational materials, with Canada's Food Guide identified as the top preference.

Perceptions on the availability of FFs were assessed by asking how easily available the FFs they were interested in consuming were. Responses varied, with 38.0% reporting “not available” and 18.6% reporting easily available (Figure 3A). Of the 9.0% that reported making FFs at home, yogurt and fermented vegetables were most often indicated (Figure 3B; Table S5). A total of 36.0% of participants indicated no interest in home fermentation, while 30.0% would make FFs at home if they knew how to (Figure 3C; Table S6). Few people consulted resources for home fermentation (30.9%), with 16.0% listing “other”, and 12.0% using university resources (Figure 3D). Nearly half of participants showed interest in receiving more information on FFs in Canada's Food Guide and one third wanted expert developed websites (32.5%) (Figure 3E). A total of 1334 participants reported a family history of making FFs, which was most common among younger individuals, those with lower BMI, higher education, urban residence, and greater health consciousness (Table S7). Additionally, six in ten participants (63.7%) indicated willingness to try eating FFs, with females and younger individuals expressing the most openness (mean = 46.1 years, *p* < 0.0001) (Figure 3C; Table S8).

## 4 Discussion

This survey of 4045 Canadian adults provides the largest assessment of FF consumption, perceptions, and knowledge among Canadian adults. Although most participants reported having consumed all the surveyed FFs at least once, intake was concentrated in fermented dairy foods such as cheese and yogurt. In contrast, fermented vegetables, cereals, plant‐based products, or fermented fish were consumed less frequently. Taste emerged as the primary driver of FF consumption, followed by perceived health benefits, whereas cultural traditions played a comparatively minor role. Despite widespread consumption of FFs, uncertainty regarding their health benefits, safety, and knowledge of fermentation practices was limited, highlighting important knowledge gaps and opportunities for evidence‐based education.

Similar patterns have been reported internationally. Surveys from Slovenia, the Netherlands and Türkiye consistently show fermented dairy products such as yogurt and cheese dominate consumption, whereas fermented vegetables, kombucha, and other products are consumed less frequently or seasonally (Ganiyusufoglu et al. 2022; Li et al. 2020; Šikić‐Pogačar et al. 2022). Together, these findings suggest that while FFs are widely incorporated into modern diets, consumption is generally limited to a small number of familiar products despite the much broader diversity of FFs available. This pattern highlights an opportunity to increase awareness and acceptance of less commonly consumed products with potential nutritional, cultural, and culinary value.

### 4.1 Drivers of Fermented Food Consumption

In this Canadian survey, taste emerged as the strongest driver of FF consumption, while extrinsic, situational, and sociocultural factors were also associated with differing FF consumption patterns. These findings are consistent with the recent systematic review by García‐Barón et al., which identified intrinsic product characteristics, particularly sensory attributes such as taste, as the strongest determinants of FF acceptance across diverse populations (García‐Barón et al. 2025). Additionally, these self‐reported motivations are consistent with previous consumer studies, such as from the HEALTHFERM European study (Perez‐Cueto et al. 2024), which identified taste and health as dominant drivers, particularly among younger and more educated individuals. In the present survey, participants reported consuming FFs also due to perceived health benefits, particularly for products such as kefir, kombucha, and plant‐based alternatives, suggesting that health perceptions may influence consumer choice differently across FF categories. This is consistent with prior reports showing that health‐oriented consumers prioritize probiotic and bioactive properties over sensory qualities (Conti‐Silva and de Souza‐Borges 2019; García‐Barón et al. 2025).

Sociodemographic characteristics such as age, gender, and education, along with cultural familiarity and generational exposure, showed associations with both the diversity and frequency of intake. Younger adults and those with higher education reported greater consumption, whereas older adults consumed fewer FFs. These differences may reflect differences in exposure to health trends, internet use, or access to a wide range of options. This is not surprising since there is research showing that dietary diversity often declines with age and is associated with health outcomes in older adults (Cano‐Ibáñez et al. 2019; Reedy et al. 2014; Rezaei et al. 2024). Click or tap here to enter text.

Household composition and urban residency were also associated with consumption patterns, suggesting social context and access to a wider variety of food options may shape FF intake. While cultural traditions were not the primary driver of FF intake, participants with a family history or cultural tradition of consuming FFs tended to report a broader variety of products consumed. Notably, first‐generation immigrants were more likely to consume traditional FFs from their countries of origin, which is in line with previous research showing the role of early‐life exposure and cultural familiarity in shaping dietary behavior (Lindsey 2024). This is particularly relevant in Canada, where over 23% of the population is foreign‐born and multicultural influences are deeply embedded in determining national dietary patterns (Statistics Canada 2021). Conversely, second‐ and third‐generation Canadians primarily consumed Western staples like yogurt, cheese, and sourdough bread, possibly reflecting a generational shift and assimilation of dietary choices. These patterns suggest potential contributions of cultural familiarity, generational exposure, and demographic characteristics on FF consumption. Consistent with these findings, García‐Barón et al. (2025) previously reported that the most influential sub‐factors affecting FF consumption were attitudes and consumption habits, followed by cultural influences and beliefs (García‐Barón et al. 2025). Additionally, a similar survey in Slovenia, reported that people regularly consumed FFs due to tradition despite limited knowledge of fermentation (Šikić‐Pogačar et al. 2022). This means that in countries where FFs are not deeply embedded in cultural traditions, awareness of their health benefits and safety may play a key role in influencing consumption.

Age, gender, education, and income also varied across patterns of consumption and preparation of FFs in our participants. Our survey indicated that females tended to show more openness to trying new FFs, consumed a higher number of products, and reported to make FFs at home more than males. Although the reasons for these differences remain unclear, previous studies have reported gender‐specific differences in dietary behaviors, taste preferences and sensitivity, as well as greater nutrition knowledge and interest in healthy eating among women (Lombardo et al. 2023; Sichert‐Hellert et al. 2011; Spinelli et al. 2025). Click or tap here to enter text. Click or tap here to enter text. Together, these factors may contribute to the greater engagement with FFs observed among female participants.

In our survey, some participants indicated placing importance on health when making food choices also tended to consume a greater variety of fermented foods, suggesting that health consciousness may be associated with fermented food consumption. Previous evidence has similarly suggested that health consciousness and the perceived nutritional value of foods are important drivers of food consumption (Wardle et al. 2004). For instance, an acceptability study of sheep milk–based yogurt found that greater interest in the nutritional and health attributes of yogurt was associated with a higher likelihood of product acceptance, alongside demographic factors (De Devitiis et al. 2023).

In this Canadian survey, age emerged as one of the strongest demographic predictors of FF consumption. Older participants showed lower consumption of FFs and tended to show less willingness to try new products. Age‐related changes in sensory perception, such as sweetness tolerance or flavor intensity, have also previously been reported to play a role in shaping acceptance of products like yogurt or fermented vegetables (Rutkowska et al. 2022; Saint‐Eve et al. 2016). Notably, while younger consumers often express positive attitudes toward FFs in the literature and in our survey, these do not always translate into purchasing behaviors as product familiarity, sensory characteristics, accessibility, and habitual dietary practices continue to strongly influence food choices (Chezan et al. 2022; García‐Barón et al. 2025; Perez‐Cueto et al. 2024).

Education and income were also positively associated with FF consumption in our Canadian survey. Participants with lower education and income consumed fewer FFs overall, whereas those with higher education were more likely to prepare FFs at home and showed greater willingness to try new FFs. These findings are consistent with previous studies demonstrating that individuals with higher educational attainment and income are more likely to adopt novel FFs and perceive greater value in their nutritional and health‐promoting properties (De Devitiis et al. 2023; Sajdakowska et al. 2021). In the literature, higher education has also been associated with greater awareness of product quality, traditional food values, and food‐related knowledge, while higher‐income consumers are more likely to seek out premium or artisanal fermented products that they perceive as healthier and more sustainable (Adinsi et al. 2014; Freschi et al. 2023; Rojas‐Rivas et al. 2020). Moreover, in our survey, residence location, marital status, and occupation were also associated with patterns of FF consumption, which is consistent with other studies in the literature (Arora et al. 2021; De Devitiis et al. 2023; García‐Barón et al. 2025; Sikombe et al. 2023). Such differences may reflect variation in cultural food traditions, access to fermented products, purchasing habits, and lifestyle factors that influence food choices.

Additionally, only 9% of participants reported preparing FF at home. This is somewhat consistent with findings from a Slovenian study, where less than one‐third (29.8%) of participants attempted to prepare FFs at home (Šikić‐Pogačar et al. 2022). In our Canadian survey, females, respondents with higher education, higher income level, and following a plant‐based or non‐omnivorous diet were also associated with responding yes to making FFs. Analyses from Statistics Canada spanning 1986–2015 indicate that women remain more likely than men to be primarily responsible for meal preparation (47.5% vs. 16.1% of households), despite a gradual increase in shared household responsibilities over time (Statistics Canada 2017, 2021). Similar patterns have been reported internationally, with women consistently undertaking meal planning, food shopping, and home cooking more frequently than men across diverse cultural settings (Lombardo et al. 2023; Storz et al. 2022). Together, these findings suggest that greater involvement in meal preparation, combined with higher education, income, and health‐conscious dietary patterns, may increase familiarity with food preparation techniques, including fermentation. Consistent with this interpretation, prior research exists indicating that higher socioeconomic status and a strong interest in health are key predictors of food preparation practices such as fermentation (Méjean et al. 2017).

Together, these findings suggest that consumer engagement with FFs is shaped by a complex interplay of sociodemographic characteristics, health‐related attitudes, and product‐specific perceptions. Although the present study did not evaluate health outcomes, these differences may influence dietary diversity and exposure to FFs across Canadian populations. Recognizing the differences in FF intake across population characteristics may help inform future public health messaging and product development strategies aimed at diversifying FF intake in Canadian populations.

### 4.2 Knowledge Gaps and Barriers of Adoption

The present survey identified knowledge gaps in participants' understanding of FFs, particularly regarding their microbial nature. Participants also reported a lack of accessible and trustworthy information on home fermentation practices, associated health benefits, and food safety. Similar confusion was observed in the HEALTHFERM project, which spanned nine European countries and identified widespread uncertainty, especially concerning plant‐based FFs (Perez‐Cueto et al. 2024), and in Slovenia fewer than 20% of participants were able to accurately define fermentation (Šikić‐Pogačar et al. 2022).

While most participants reported good access to FFs, the lack of clear public guidance, education, and regulation might contribute to ongoing consumer confusion and uncertainty. The relevance of these concerns is highlighted by findings from a recent survey of Canadian university students. Although 78% of participants believed they were familiar with the term “fermented foods,” only 23% could accurately define fermentation when asked to describe the process (Hekmat and Ahmadi 2025). Even students enrolled in Food and Nutrition programs answered knowledge questions correctly only 67% of the time (compared with 62% among students from other disciplines Hekmat and Ahmadi 2025). These results suggest that gaps in understanding are widespread and persist even among those training for nutrition‐related professions. Such evidence reinforces the concern that limited coverage of fermentation within dietetics education and national nutrition guidance leaves both future professionals and the public underinformed.

### 4.3 Trends and Opportunities

Our survey findings revealed several emerging trends and opportunities related to FF consumption in Canada. Younger adults, women, and individuals with higher education and income levels expressed greater openness to trying new or unfamiliar fermented products, suggesting they may serve as early adopters for novel FFs. Products like kefir, kimchi, and kombucha were often cited as appealing by some and off‐putting by others due to their flavor or smell. This underscores an opportunity for the functional food sector to develop more palatable versions of FFs that confer health benefits while appealing to broader sensory preferences. Reformulation strategies, improved packaging, and targeted messaging could support wider adoption (García‐Barón et al. 2025; Saint‐Eve et al. 2021). Moreover, our findings also highlight an opportunity for the food industry to strengthen the scientific evidence supporting the health effects of these products. Investment in well‐designed clinical trials evaluating specific FFs could help substantiate potential health benefits, improve consumer confidence, and ultimately facilitate wider adoption.

Despite the widespread availability of fermented products in Canada, these survey findings reveal gaps in consumer understanding of FFs. Many participants were uncertain about the role of microbes in fermentation, the safety of these foods, and how they differ from probiotics. Knowledge of specific health benefits was also limited. These findings suggest that consumption of FFs does not necessarily translate into an understanding of their production or potential health effects. Addressing these knowledge gaps represents an important opportunity for nutrition education and public health initiatives.

Additionally, nearly one‐third of participants expressed interest in home fermentation if provided with appropriate tools and guidance, suggesting that consumer engagement could be increased with education and public health initiatives. Although home fermentation remains a niche practice, it represents an opportunity to increase dietary diversity, preserve cultural food traditions, and improve food literacy. This is supported by other research showing that greater involvement in food preparation is associated with healthier dietary patterns, including higher intakes of fruits, vegetables, and dietary fiber (Larson et al. 2006). The interest in home fermentation among younger and health‐conscious consumers observed in our survey suggests there is potential for this practice to expand alongside growing consumer interest in minimally processed foods, sustainable food practices, and gut health. However, realizing this potential will require greater public confidence in the safety and practical aspects of home fermentation too. In addition, clear labeling and accessible resources may assist in reducing barriers and building confidence toward home fermentation.

Additionally, national dietary guidelines serve as a primary source of nutrition information for the public. This was reflected in the survey responses, where many participants indicated that the Food Guide was their preferred place to access information about fermented foods. However, Canada's Food Guide does not explicitly address FFs, typically referencing a narrow set of examples such as dairy products (Chilton et al. 2015). This under‐representation is not unique to Canada; similar gaps appear in other national dietary guidelines (Chilton et al. 2015; Li et al. 2020). From a public health perspective, these findings highlight opportunities to improve consumer understanding of fermented foods through evidence‐based education and clearer communication regarding their production, safety, and potential health effects. Continued strengthening of the clinical evidence for specific fermented foods will also be important to support future evidence‐based dietary recommendations and informed consumer choices.

Moreover, fermentation supports environmental sustainability by extending shelf‐life, reducing food waste, and supporting local production (Dhiman et al. 2025; Gänzle et al. 2024). Additionally, microbial‐based food processing is less dependent on climate and seasonality, which can offer reliance under environmental stress (Graham and Ledesma‐Amaro 2023). Canada's culturally diverse population and strong agriculture sector further position the country well to advance research, innovation, and evidence‐informed policies in this field.

### 4.4 Study Limitations

Although this study included a large, population‐based sample designed to reflect Canadians, several limitations should be acknowledged. In addition to participants through Qualtrics participant database, researchers distributed this survey to colleagues and fellow researchers which may introduce bias in participants, as those researchers tend to be aware of the health impacts of FFs. Those who received the survey from Qualtrics may also have been more likely to participate if they were already interested in nutrition or health, which is a limitation of this survey and distribution method. Furthermore, because recruitment relied primarily on online methods, individuals without reliable internet access or those less likely to participate in online surveys may have been underrepresented, introducing the potential for selection bias. In addition, self‐reported intake is subject to recall and social desirability bias, where an individual may introduce bias by being led by the question to report what they believe is the correct answer, or by being unable to recall information correctly due to time elapsed from food consumption to survey completion. Social desirability bias was mitigated by using neutral, non‐leading language throughout the survey; however, self‐reported dietary intake remains subject to reporting error, and recall bias may be present for the foods consumed section as there was not a time period that these foods were required to be consumed in and instead could have been at any point in their lifetime. Without this time limit individuals may have reported foods consumed only infrequently or historically, possibly leading to an overrepresentation of foods rarely consumed rather than staples in diets. The survey question on perceptions of FFs asked whether they are “good for you,” rather than whether they “confer health benefits,” leaving room for varied interpretation. Similarly, questions about safety did not distinguish between commercial and home‐fermented foods, which may have influenced responses. Finally, the question on information sources for home fermentation omitted the option of “family or friends' recipes.” Given the traditional and community‐based nature of fermentation practices, this may explain why some participants selected “None,” meaning “none of the listed options” rather than “no source at all.” Future surveys should refine question wording and response options to improve interpretability and representativeness. A cross‐sectional design such as this is also limited by being only a snapshot of the population, thus being unable to track changes over time and unable to determine causal relationships, instead identifying only associations. As such there is no way of knowing which factor influences the other, whether demographic characteristics influence FF consumption or whether FF consumption is associated with these demographic characteristics.

## 5 Conclusion

This study provides the first comprehensive assessment of FF consumption, perceptions, and knowledge among Canadian adults. Our findings demonstrate that FF consumption is shaped by a combination of sensory preferences, health‐related motivations, sociodemographic characteristics, and cultural familiarity. Despite the widespread consumption of FFs among participants, important gaps remained in their knowledge of what constitutes a FF, how fermentation occurs, the safety of these products, and their potential health effects. While the present survey cannot determine whether FF consumption improves health in this population, it provides valuable information on consumer knowledge, attitudes, and barriers that can inform future education and knowledge translation efforts. As the evidence base for FFs continues to grow, these findings may also help inform the development of educational resources and implementation strategies that address existing knowledge gaps and consumer needs. Altogether, these survey findings offer valuable direction for future research, education, and policy development.

## Author Contributions

**Samantha Tapping:** conceptualization, data curation, methodology, investigation, validation, formal analysis, visualization, writing – review and editing, writing – original draft. **Raylene A. Reimer:** conceptualization, supervision, project administration, writing – review and editing, funding acquisition. **Kara Sampsell:** writing – original draft, writing – review and editing, methodology, conceptualization, data curation, investigation. **Carla Liria Sánchez‐Lafuente:** data curation, formal analysis, visualization, project administration, resources, writing – original draft, writing – review and editing. **Camila Schultz Marcolla:** conceptualization, writing – original draft, writing – review and editing, visualization, formal analysis, data curation, methodology, validation, investigation. **Gabrielle Eaton:** visualization, writing – original draft, writing – review and editing. **Benjamin P. Willing:** conceptualization, project administration, supervision, writing – review and editing, funding acquisition. **Ellen Goddard:** data curation, formal analysis, validation. **Jeremy P. Burton:** conceptualization, supervision, project administration, writing – review and editing, funding acquisition.

## Funding

This work was supported by the Weston Family Foundation, Phase I & II.

## Conflicts of Interest

The authors declare no conflicts of interest.

## Supporting information


**Data S1:** Supporting Information.

**Table S1:** Survey used in the study.
**Table S2:** Knowledge of fermentation process by demographic characteristics. *N* (%).
**Table S3:** Knowledge of FF definition by demographic characteristics. *N* (%).
**Table S4:** Perceived safety and knowledge of fermented foods by demographic characteristics. *N* (%).
**Table S5:** Distribution of responses toward making fermented foods at home by demographic factors. *N* (%).
**Table S6:** Willingness to try making fermented foods at home by demographic characteristics. *N* (%).
**Table S7:** Family history of making FFs at home by demographic characteristics. *N* (%).
**Table S8:** Openness to trying fermented foods by demographic characteristics. *N* (%).
**Table S9:** Correlation matrix statistics.
**Table S10:** Thematic categorization of open‐ended responses on Q29 regarding perceived healthfulness of fermented foods. Responses were manually categorized and could be assigned to more than one theme when multiple concepts were expressed; therefore, category counts are not mutually exclusive and should not be summed to obtain the total number of respondents.

## Data Availability

The data that support the findings of this study are available from the corresponding author upon reasonable request. The survey instrument and additional tables and figures are available in the Supporting Information.
