# Supplementary material for: Consumer Perceptions and Behaviors Toward Fermented Foods: Insights From a Canadian Survey
Source: Food Sci Nutr. 2026 Aug 28;14(9):e72287. doi: 10.1002/fsn3.72287 (PMC13522835; doi:10.1002/fsn3.72287)
Supplement: Supplementary file 1 [file FSN3-14-e72287-s001.docx]

**Data processing and handling**

**General cleaning and preprocessing:**

The dataset required cleaning prior to any analysis taking place. This included removal of unnecessary rows and columns, such as the wording of the questions and the time taken to complete the survey.

The remaining processing was done on a question-by-question basis.

**Q1. What is your current age in years**

The age was both left as a continuous variable that was analyzed as well as a new variable created called, age_ranked, which split the ages into 6 categories.

| **Age** | **Age_Ranked** |
| --- | --- |
| 18-25 | 1 |
| 26-35 | 2 |
| 36-45 | 3 |
| 46-55 | 4 |
| 56-65 | 5 |
| 66+ | 6 |

**Q2. In which Canadian province or territory do you live? If you do not currently live in a Canadian province or territory, please select the option the best describes your situation.**

The provinces were left as separate entities but were encoded to shorter lengths so that there were no spaces, to allow for cleaner analysis downstream.

| **Province** | **Province** |
| --- | --- |
| British Columbia | BC |
| Newfoundland and Labrador | NAL |
| New Brunswick | NB |
| Nova Scotia | NS |
| Northwest Territories | NWT |
| I am a resident (temporary or permanent) or citizen of Canada that is currently living outside of Canada | Outside |

**Q3. What is your sex?**

The people who left the question blank had that blank changed to the word “Unknown”.

**Q4. The weight of the participants.**

The participants were allowed enter their weight in either pounds or kilograms. These values needed to be standardized, and the following conditions were used to do so. If the participant left both columns black their response was set to “Unknown”. If they filled out just the section for pounds and the value they entered was less than 100, it was assumed the weight was in kilograms and was left as is, if the value entered was more than 100 it was assumed to be in pounds and was converted to kilograms. If they filled out just the section for kilograms then it was kept as is. If both columns were filled out then it was checked to see if the kilogram measurement was less than 40, if it was then the pound measurement was converted and used. Lastly, any values outside the boundaries of the 5^th^ to 95^th^ percentile were set to “Unknown” as they were likely outliers.

**Q5. The height of the participants.**

The participants were allowed to enter their weight in either inches or centimeters. If they filled out neither option the blank was changed to “Unknown.”

If the participant only filled out the inches, then the value inside was checked. If that value was greater than 100, it was assumed that the value was in centimeters, if the value was less than 10, it was assumed that the value provided was in feet, with the format x.y where x is feet and y is inches, and this was then converted to centimeters. If only the inches column was filled out but neither of the previous two criteria were met then it was assumed that it was in inches and was converted to centimeters.

If only the column for centimeters was filled out and it was greater than 100 it was assumed it was in centimeters and was left as is. However, if it was less than 2 then it was assumed it was in meters, and was converted to centimeters. If only the centimeters column was filled out but was between 2 and 10 it was assumed that this value was in feet and was calculated the same as above. If only the centimeters column was filled out and was between 10 and 100 it was assumed to be in inches and was converted to centimeters.

If both columns were filled out and were both less than 12, it was assumed that the participant gave their height in feet and inches, with the first column being feet and the second column being inches, their height was converted to centimeters. If both columns were filled out, with the inches column between 3 and 10, and the centimeters column being less than 135 it was assumed to be in feet and inches with the format x.y and was converted to centimeters using the above methodology. If both columns were filled out and the column for inches was between 55 and 100 it was assumed to be in inches and was converted to centimeters. If both columns were filled out but none of the above conditions were met, it was assumed to be in centimeters and had no further processing performed.

Any values outside of the 2.5^th^ percentile and 97.5^th^ percentile were set to “Unknown”.

From the now processed weight and height the BMIs were calculated when both measurements were present. If either or both measurements were missing the BMI was set to “Unknown”

**Q5. What is your highest level of education?**

The responses to this question were shortened to remove spaces allowing easier processing in R downstream.

| **Before** | **After** |
| --- | --- |
| University (undergraduate) | Undergrad |
| University (professional/post-graduate/doctoral) | Post |
| Elementary to high school | EtoHS |
| Community college | CC |
| Trade school | TS |
|  | Unknown |

**Q7. What is your average household income in dollars**

The responses to this question were shortened to remove spaces allowing easier processing in R downstream.

| **Before** | **After** |
| --- | --- |
| < 30,000 | 1 |
| 30,000 – 50,000 | 2 |
| 51,000 – 80,000 | 3 |
| 81 – 120,000 | 4 |
| > 120,000 | 5 |
|  | Unknown |

**Q8. What is your marital status?**

The responses to this question were shortened to remove spaces allowing easier processing in R downstream.

| **Before** | **After** |
| --- | --- |
| Single (never legally married) | Single |
| Common-law | CL |
|  | Unknown |

These responses were also grouped for certain analyses.

| **Marital_status** | **Marital_status_ranked** |
| --- | --- |
| Single, divorced, separated, widowed | 1 |
| Married, CL | 2 |
| Unknown | 3 |

**Q9. How many people live in your household?**

The responses to this question were shortened to remove spaces allowing easier processing in R downstream.

| **Categorical** | **Rank** |
| --- | --- |
| 1 (I live alone) | 1 |
| 2 to 4 | 2 |
| More than 4 | 3 |
|  | Unknown |

**Q10. How many children for whom you are the parent/legal guardian live with you in your household?**

The responses to this question were shortened to remove spaces allowing easier processing in R downstream.

| **Categorical** | **Rank** |
| --- | --- |
| None | 0 |
| 1 | 1 |
| 2 to 4 | 2 |
| More than 4 | 3 |
|  | Unknown |

**Q11. Which ethnicity(s), race(s), and/or culture(s) do you identify as? (Select all that apply)**

Over 2/3 of the individuals who listed a race selected “white,” for this reason race was excluded for future analysis.

**Q12. How many generations of your family have lived in Canada?**

Urban represented 42.5% of the answers and was considered as the reference category for OLS. No missing data.

| **Before** | **After** |
| --- | --- |
| 3 or more | 3 |
|  | Unknown |

**Q13. What best describes the area where you live?**

A new column was added to rank the living areas.

| **Categorical** | **Rank** |
| --- | --- |
| Urban | 1 |
| Suburban | 2 |
| Rural | 3 |

**Q14. Do you consider yourself healthy?**

The answers were shortened to remove spaces for downstream analysis.

| **Before** | **After** |
| --- | --- |
| Not sure | NS |
|  | Unknown |

**Q15. How many times per year do you see your family doctor?**

The answers were shortened to remove spaces for downstream analysis.

| **Before** | **After** |
| --- | --- |
| 2 to 4 | 2 |
| 5 or more | 3 |

**Q16. What best describes how frequently you consider health benefits when you choose food to eat?**

The answers were shortened to remove spaces for downstream analysis.

| **Before** | **After** |
| --- | --- |
| Never | 1 |
| Sometimes | 2 |
| Most of the time | 3 |
| Always | 4 |

**Q17. What best describes how frequently you consider health benefits when you choose food to eat?**

The answers were shortened to remove spaces for downstream analysis.

| **Before** | **After** |
| --- | --- |
| Omnivorous (I eat both animal and plant-based foods, including meat) | Omnivorous |
| Pescatarian (I eat fish and plant foods but do not eat meat; I may or may not eat eggs and/or milk products) | Pescatarian |
| Vegetarian (I eat plant foods but do not eat meat or fish; I may or may not eat eggs and/or milk products) | Vegetarian |
| Vegan (I only eat plant-based foods. I do not eat meat, fish, or any animal products) | Vegan |
|  | Unknown |

**Q18 and 19. Fermented foods consumed, frequency of consumption, and reason for consumption.**

Questions 18 and 19 were reformatted so that instead of a list of foods there was now a “yes/no” column for if each food option in the survey was consumed, a column for the frequency of consumption of that food, and a column indicating the primary reason for consumption of each food.

The free response option for “Other” in the reasoning was recording and false positives were removed, where a person indicated that “Yes” they had eaten the food, but their free response indicated they had not, this was then changed to indicate they had not eaten the food in question.

For both the frequency column and primary reason for consumption column the answers were simplified to remove spaces for downstream analysis.

| **Frequency** | **Eaten_x_freq** |
| --- | --- |
| I have consumed this food | 0 |
| 1 time per month or less, 2-3 times per month | 1 |
| 1-2 times per week, 3-4 times per week, 5-6 times per week | 2 |
| 1 time per day, 2-3 times per day, 4 or more times per day | 3 |
| Not consumed | NA |
| Multiple options selected | Unknown |

| **Reason for consumption** | **X_reason** |
| --- | --- |
| The product tastes good | Taste |
| Promote overall health | Health |
| Manage a disease or condition | Condition |
| Cultural or heritage/tradition reasons | Cultural |
| Other (please enter): input box | Other |

Lastly a column was added which contained the sum of the fermented foods eaten by a participant from the options provided.

**Q20. Do you consume non-heated (non-pasteurized) fermented foods such as non-heated fermented vegetables (e.g. non-heated kimchi or non-heated sauerkraut) or non-heated sausage (e.g. non-heated salami)?**

The answers were shortened to remove spaces for downstream analysis.

| **Before** | **After** |
| --- | --- |
| Not sure | NS |
|  | Unknown |

**Q21. Do you make fermented foods at home?**

The free responses for this question had the participants put in a list of fermented foods they made at home; these were categorized to match the categories from question 18 with any others being added to a new category. Each category got its own yes/no column for if that participant made that food. The original column for if they make fermented foods at all was kept with the following changes, to remove spaces.

| **Before** | **After** |
| --- | --- |
| Yes [please enter the foods separated by a comma (e.g. yogurt, kimchi)]: | Yes |
|  | Unknown |

A new column was added to the table as well, which was the total number of fermented foods that each individual made at home.

**Q22. If you have children in your household, do they eat fermented foods?**

The free responses for this question had the participants put in a list of fermented foods they made at home; these were categorized to match the categories from question 18 with any others being added to a new category. Each category got its own yes/no column for if that participant made that food. The original column for if they make fermented foods at all was kept with the following changes, to remove spaces. If a participant indicated in question 10 had no children, their rows were set to NA.

| **Before** | **After** |
| --- | --- |
| Yes [please enter the foods separated by a comma (e.g. yogurt, kimchi)]: | Yes |
|  | Unknown |

A new column was added to the table as well, which was the total number of fermented foods that each individual made at home.

**Q23. What type of resources do you use as sources of information on how to ferment foods at home, if any?**

A yes/no column was created for each checkbox option from the question, with each selected option for the participants being a “yes,” The unique text responses for “other” were stores in a separate document

**Q24. Would you make fermented foods if you knew how to prepare them?**

The answers were shortened to remove spaces for downstream analysis.

| **Before** | **After** |
| --- | --- |
| Not sure | NS |
| I already make fermented foods at home | Already |
|  | Unknown |

**Q25. Fermented food availability.**

The answers were shortened to remove spaces for downstream analysis.

| **Before** | **After** |
| --- | --- |
| Easily available | 4 |
| Mostly available | 3 |
| Somewhat difficult to find | 2 |
| Not available | 1 |
| I do not purchase fermented foods | 0 |
|  | Unknown |

**Q26. Is there a history of fermented foods in your culture?**

The answers were shortened to remove spaces for downstream analysis.

| **Before** | **After** |
| --- | --- |
| Not sure | NS |
|  | Unknown |

**Q27. Did older generations in your family make fermented foods?**

The answers were shortened to remove spaces for downstream analysis.

| **Before** | **After** |
| --- | --- |
| Not sure | NS |
|  | Unknown |

**Q28. Do you like the taste of fermented foods?**

The answers were shortened to remove spaces for downstream analysis.

| **Before** | **After** |
| --- | --- |
| Not sure | NS |
|  | Unknown |

**Q29. Do you think fermented foods are good for you?**

A column to hold just the participants base responses was created and had answers simplified.

| **Before** | **After** |
| --- | --- |
| Not sure | NS |
| Yes [please specify primary reason(s)]: | Yes |
|  | Unknown |

The participants free responses were then categorized in a separate document based on their primary reason(s) for consumption. They were separated into categories for gut health, microbes, reasons these individuals did not eat fermented foods, apprehensions and confusions around fermented foods, dietary reasons, other reasons, and that they did not consume fermented foods. A response could fit into multiple categories, and any false positives identified through this method were changed to negative responses.

**Q30. Are you open to trying new fermented foods?**

The answers were shortened to remove spaces for downstream analysis.

| **Before** | **After** |
| --- | --- |
| Not sure | NS |
|  | Unknown |

**Q31. What factors least appeal to you about trying new fermented foods?**

As a multiple select question, each option got a yes/no column to indicate if the participant found that particular factor unappealing. The free responses were stored in a separate document.

**Q32. Do you perceive probiotics and fermented foods to be the same thing?**

The answers were shortened to remove spaces for downstream analysis.

| **Before** | **After** |
| --- | --- |
| Not sure | NS |
|  | Unknown |

**Q33. Microorganisms (e.g. bacteria, yeast) are used to make fermented foods.**

The answers were shortened to remove spaces for downstream analysis.

| **Before** | **After** |
| --- | --- |
| Not sure | NS |
|  | Unknown |

**Q34. Fermented foods are completely safe.**

The answers were shortened to remove spaces for downstream analysis.

| **Before** | **After** |
| --- | --- |
| Not sure | NS |
|  | Unknown |

**Q35. What educational materials would you prefer to use to access more information about fermented foods?**

As a multiple select question, each option got a yes/no column to indicate if the participant would want that educational material to have fermented foods included. The free responses were stored in a separate document.

**Processing for descriptive statistics:**

Unknowns were kept in for any tables generated, but were removed from any statistical testing performed.

**Processing for regression model:**

**Q1. What is your current age in years**

For OLS regression model, age (in years) was kept as a numeric, continuous variable. For visualization, age was ranked into the following numeric categories.

| **Age** | **Age_Ranked** |
| --- | --- |
| 18-25 | 1 |
| 26-35 | 2 |
| 36-45 | 3 |
| 46-55 | 4 |
| 56-65 | 5 |
| 66+ | 6 |

**Q2. In which Canadian province or territory do you live? If you do not currently live in a Canadian province or territory, please select the option the best describes your situation.**

For OLS regression model, the answers for provinces were combined according to region, reducing the number of dummy variables from 13 to 5. Central Canada had 56.2% of the respondents and was used as the reference category. Canadian citizens living outside of Canada represented 1.4% of the data and were combined to Central Canada for statistical analysis.

| **Provinces** | **Region (dummy variable)** |
| --- | --- |
| Newfoundland and Labrador, Prince Edward Island, Nova Scotia, New Brunswick | **The Atlantic Region** (Atlantic) |
| Quebec, Ontario | **Central Canada** (Central) |
| Manitoba, Saskatchewan, Alberta | **The Prairie Provinces  (Prairies)** |
| British Columbia | **The West Coast**  (West) |
| Nunavut, Northwest Territories, Yukon Territory | **The North (North)** |
| Outside of Canada (1.4%) | **Combined to reference category** |

**Q3. What is your sex?**

For OLS regression model, the reference category used was Females (51.7% of the respondents). For statistical analysis, people that did not answer the question (“Unknown”, 0.1%) were combined to the reference category. “Other” was 0.4% of the data.

**Q6. What is your highest level of education?**

The categorical variables provided were re-coded into corresponding years of education for OLS analysis as follows. Answers that were not provided (“Unknown, 0.3%) were transformed into the average (14 years).

| **Categorical** | **Numerical** |
| --- | --- |
| Elementary to high school | 12 |
| Trade school and undergraduate | 14 |
| Graduate | 16 |
| Postgraduate | 18 |

**Q7. What is your average household income in dollars**

The categorical answers for income were transformed to numerical variables using the mid-point from each answer provided. Missing data (0.2%) were assigned to the 65,000 range.

| **Categorical** | **Rank** | **Numerical** |
| --- | --- | --- |
| < 30,000 | 1 | 15,000 |
| 30,000-50,000 | 2 | 40,000 |
| 51,000-80,000 | 3 | 65,000 |
| 81,000-120,000 | 4 | 100,000 |
| >120,000 | 5 | 120,000 |

**Q8. What is your marital status?**

The categorical answers for marital status were combined into dummy variables for OLS analysis. Missing data (0.1%) was added to the reference category. The reference category was “Married”, which included both “married” and “common-law” status.

| **Categorical** | **Dummy** |
| --- | --- |
| Single | Single |
| Married, Common-law | Married |
| Divorced/Separated | Divorced |
| Widowed | Widowed |

**Q9. How many people live in your household?**

The categorical answers for household size were ranked for OLS analysis. Missing data Missing data (0.1%) was added to the reference category. The reference category was “2 to 4”, representing 68.3% of the data.

| **Categorical** | **Rank** |
| --- | --- |
| 1 (I live alone) | 1 |
| 2 to 4 | 3 |
| More than 4 | 5 |
| Unknown | 3 |

**Q10. How many children for whom you are the parent/legal guardian live with you in your household?**

The categorical answers for presence of children were ranked for OLS analysis. Missing data (0.1%) was added to the reference category. The reference category was “None”, representing 66.7% of the data.

| **Categorical** | **Rank** |
| --- | --- |
| None | 0 |
| 1 | 1 |
| 2 to 4 | 3 |
| More than 4 | 5 |
| Unknown/Missing | 0 |

**Q11. Which ethnicity(s), race(s), and/or culture(s) do you identify as? (Select all that apply)**

People that selected “white” represented 66.6% of the data and white was used as the reference category for OLS analysis. Due to the complexity of answers and the low proportion of respondents within other ethnicities and races, answers were combined into “non-white” category for OLS analysis.

**Q13. What best describes the area where you live?**

Urban represented 42.5% of the answers and was considered as the reference category for OLS. No missing data.

**Q12. How many generations of your family have lived in Canada?**

Missing data (0.2%) was assigned to 3 or more (50.4%)

**Q26. Is there a history of fermented foods in your culture?**

Reference is NO (45.2%), missing data (0.2%) was added to reference.

**Q27. Did older generations in your family make fermented foods? (e.g. your parents, grandparents etc.)**

Reference is NO (42.6%), missing data (0.2%) was added to reference.

**Q4 & Q5. Please enter your weight and height.**

These questions were combined to calculated BMI. Every BMI that could not determined (n= 685, 17%) was changed into 27.09 (mean BMI) for the dataset.

**Q14. Do you consider yourself healthy?**

Reference is YES (75.6%), missing data (0.02%) was added to reference.

**Q15. How many times per year do you see your family doctor?**

The categorical answers were ranked for OLS analysis. There was no missing data.

| **Categorical** | **Rank** |
| --- | --- |
| 0 | 0 |
| 1 | 1 |
| 2 to 4 | 3 |
| 5 or more | 5 |

**Q16. What best describes how frequently you consider health benefits when you choose food to eat?**

Categorical answers were ranked for OLS analysis. There was no missing data.

| **Categorical** | **Rank** |
| --- | --- |
| Never | 0 |
| Sometimes | 1 |
| Most of the time | 2 |
| Always | 3 |

**Q17. What dietary pattern best describes the way that you eat?**

For OLS analysis, omnivorous (89.1%) was kept as the reference category. Missing data (4.3%) was assigned to the reference.

**Q18. Please check the box next to each of the fermented foods that you have consumed.** **If you currently consume the food, please also select a second box to tell us whether you consume it 1 time per month or less, 2-3 times per month, 1-2 times per week, 3-4 times per week, 5-6 times per week, 1 time per day, 2-3 times per day, 4 or more times per day. If you do not eat the food, then you do not need to select any boxes.**

Preliminary analyses demonstrated substantial clustering at the upper limit of the number-of-products variable, with approximately 30% of respondents reporting consumption of all listed fermented food categories.

The categorical answers were ranked. If more than one frequency was selected, only the highest frequency was kept. This question was considered as an outcome variable in the OLS model (model 1) as it was used to calculate consumption score.

| Never | 0 |
| --- | --- |
| I have consumed this food | 1 |
| Once a month | 2 |
| 2-3 times per month | 3 |
| 1-2 times per week | 4 |
| 3-4 times per week | 5 |
| 5-6 times per week | 6 |
| 1 time per day | 7 |
| 2-3 times per day | 8 |
| 4 or more times per day | 9 |

**Consumption score:** a new variable called consumption score was created by summing the frequencies of consumption of each product and the total number of products consumed aiming at measuring overall exposure to fermented food products.

The consumption score distribution looks like this:

Scores go from 0 to 180, 180 being equal to consuming the 18 products 3-4 times a day (162+18). Only 2 respondents scored 180. This improved data distribution, as considering only the total number of products consumed skews the data (30% of the respondents would have consumed all the products). Average score for the dataset is 42. Highest score excluding 180 is 165.

By using consumption score, each products contributes equally to the calculation, in that way, a person that consumes all products once a month (18*2=36) has a similar score than someone who consumes only 4 products, but with 2-3 times a day frequency (4*8= 32)

**Q19. Please indicate the primary reason that you have consumed each of the fermented foods that you selected in Q18**

The answers, including missing data (Unknown) were transformed into dummy variables. This question was not considered in the OLS model (model 1) that had consumption score as an outcome variable.

**Q20. Do you consume non-heated (non-pasteurized) fermented foods?**

Reference is NO (45.6%), missing data (0.1%) was added to reference.

**Q21. Do you make fermented foods at home?**

Reference is NO (45.6%), missing data (0.1%) was added to reference.

**Q22. If you have children in your household, do they eat fermented foods?**

Not added to the model.

**Q23. What type of resources do you use as sources of information on how to ferment foods at home, if any?**

Not added to the model.

**Q24. Would you make fermented foods if you knew how to prepare them?**

Reference is NO (36.1%), missing data (0.1%) was added to reference.

**Q25. Please choose the response that best describes the availability of the fermented foods you would like to purchase.**

Answers were ranked and missing data (0.2%) was assigned to 3.

| Answer | Rank |
| --- | --- |
| Not available | 0 |
| Not available | 1 |
| Somewhat difficult to find | 2 |
| Mostly available | 3 |
| Easily available | 4 |

**Q28. Do you like the taste of fermented foods?**

Reference is YES (60.1%), missing data (0.2%) was added to reference.

**Q30. Are you open to trying new fermented foods?**

Reference is YES (63.7%), missing data (0.2%) was added to reference.

**Q31. What factors least appeal to you about trying new fermented foods?**

Not added to the model.

**Q35. What educational materials would you prefer to use to access more information about fermented foods?**

Not added to the model.

**Q29. Do you think fermented foods are good for you?**

Reference is NOT SURE (52.5%), missing data (0.4%) was added to reference.

**Q32. Do you perceive probiotics and fermented foods to be the same thing?**

Reference is NO (40.6%), missing data (0.3%) was added to reference.

**Q33. Microorganisms (e.g. bacteria, yeast) are used to make fermented foods.**

Reference is YES (59.2%), missing data (0.3%) was added to reference.

**Q34. Fermented foods are completely safe.**

Reference is NOT SURE (45.1%), missing data (0.3%) was added to reference.
