# Supplementary material for: Consumer Perceptions and Behaviors Toward Fermented Foods: Insights From a Canadian Survey
Source: Food Sci Nutr. 2026 Aug 28;14(9):e72287. doi: 10.1002/fsn3.72287 (PMC13522835; doi:10.1002/fsn3.72287)
Supplement: Supplementary file 2 [file FSN3-14-e72287-s002.docx]

**Supplementary Tables**

**Supplementary Table 1.** Survey used in the study.

| **Letter of Information Prior to Survey Entrance (The letter of information is shown with an option to download a copy)**  *The following question provides access to the actual survey:*  If you are interested and may be eligible to complete this survey, then please indicate that you would like to access the survey below.  If not, you may exit this letter now by closing your web browser tab or selecting "I do not want to access this survey" below.  All survey responses will be anonymous to the researchers. We appreciate your time!   **Please note:** The red arrow buttons are used to navigate between pages. Please answer questions carefully as you will not be able to return to the first two survey questions to change your answers.   - I would like to access this survey now - I do not want to access this survey | |
| --- | --- |
| **Block 1: Background and Demographics** | |
| Background information  (prior to Q1) | **Purpose:** This survey explores Canadian consumer perceptions and intake of fermented foods.  **Please note:** Information collected here helps us understand the consumers of fermented foods in Canada. However, if you do not wish to answer some questions, please feel free to move on to the next question. |
| Q1  (free response box, if number entered is less than 18 then survey ends) | What is your current age in years? |
| Q2  (multiple choice: single response allowed, if last choice is selected then survey ends) | In which Canadian province or territory do you live? If you do not currently live in a Canadian province or territory, please select the option the best describes your situation.   - Alberta - British Columbia - Manitoba - New Brunswick - Newfoundland and Labrador - Nova Scotia - Ontario - Prince Edward Island - Quebec - Saskatchewan - Northwest Territories - Nunavut - Yukon - I am a resident (temporary or permanent) or citizen of Canada that is currently living outside of Canada - I do not currently live in a Canadian province or territory and I am not a Canadian citizen or permanent resident |
| Q3  (multiple choice: single response allowed) | What is your sex?   - Male - Female - Other |
| Q4  (two free response boxes: 1 for lbs, 1 for kg) | Please enter your weight in either pounds or kilograms. |
| Q5  (two free response boxes: 1 for inches, 1 for cm) | Please enter your height in either inches or centimeters. If you are unsure what your height is in inches or centimeters, you can check this table: *(link to the following table)*   |
| Q6  (multiple choice: single response allowed) | What is your highest level of education?   - Elementary to high school - Trade school - Community College - University (undergraduate) - University (professional/post-graduate/doctoral) |
| Q7  (multiple choice: single response allowed)  Q8  (multiple choice: single response allowed) | What is your average household income in dollars?   - < 30,000 - 30,000-50,000 - 51,000-80,000 - 81,000-120,000 - >120,000   What is your marital status?   - Single (never legally married) - Married - Common-law - Divorced - Separated - Widowed |
| Q9  (multiple choice: single response allowed) | How many people live in your household?   - 1 (I live alone) - 2-4 - More than 4 |
| Q10  (multiple choice: single response allowed) | How many children for whom you are the parent/legal guardian live with you in your household?   - None - 1 - 2-4 - More than 4 |
| Q11  (checkbox list: multiple responses allowed, free response for Other) | Which ethnicity(s), race(s), and/or culture(s) do you identify as? (Select all that apply)   - Indigenous - South East Asian (e.g. Philippines, Vietnam) - East Asian (e.g. Korea, Japan) - South Asian (e.g. Bangladesh, Pakistan) - West Asian (e.g. Iran, Afghanistan) - Indian - Black - White - European - Hispanic or Latina(o) - Other/not listed - Prefer not to share |
| Q12  (multiple choice: single response allowed) | How many generations of your family have lived in Canada? (Note: Choosing 1 generation would indicate that you are foreign-born and immigrated to Canada during your lifetime. Please include your own generation when answering this question)   - 1 - 2 - 3 or more |
| Q13  (multiple choice: single response allowed) | What best describes the area where you live?   - Rural - Suburban - Urban |
| Q14  (multiple choice: single response allowed) | Do you consider yourself healthy?   - Yes - No - Not sure |
| Q15  (multiple choice: single response allowed) | How many times per year do you see your family doctor?   - 0 - 1 - 2-4 - 5 or more |
| Q16  (multiple choice: single response allowed) | What best describes how frequently you consider health benefits when you choose food to eat?   - Never - Sometimes - Most of the time - Always |
| Q17  (multiple choice: single response allowed) | What dietary pattern best describes the way that you eat?   - Omnivorous (I eat both animal and plant-based foods, including meat) - Pescatarian (I eat fish and plant foods but do not eat meat; I may or may not eat eggs and/or milk products) - Vegetarian (I eat plant foods but do not eat meat or fish; I may or may not eat eggs and/or milk products) - Vegan (I only eat plant-based foods. I do not eat meat, fish, or any animal products) |
| **Block 2: Current Behaviours Around Fermented Foods** | |
| Background information  (prior to Q18) | The remainder of the survey is now focused specifically on fermented foods. This survey does not include questions about fermented alcoholic beverages like wine or beer. |
| **Question (Q) number and type of response allowed** | **Question text included in survey** |
| Q18  (Checkbox array: multiple responses, those selected will appear in Q19, if no boxes checked then skip to Q20)  *Horizontal checkbox array next to each of the foods:* | Please check the box next to each of the fermented foods that you have consumed. If you currently consume the food, please also select a second box to tell us whether you consume it 1 time per month or less, 2-3 times per month, 1-2 times per week, 3-4 times per week, 5-6 times per week, 1 time per day, 2-3 times per day, 4 or more times per day. If you do not eat the food, then you do not need to select any boxes.   - Yogurt - Kefir - Cheese - Sour cream - Other fermented milk products (e.g. villi, ayran) - Miso - Tempeh - Soy Sauce - Other fermented soy-based products (e.g. natto, gochujang, kinema) - Fermented vegetables (e.g. sauerkraut, kimchi, cortido) - Fermented sausage (e.g. salami, pepperoni) - Fermented fish (e.g. igunaq, surströmming) - Fermented fruits (e.g. green mango, green papaya) - Leavened bread - Fermented cereals or grains (e.g. ting, dosa, khambir) - Kombucha - Vinegar (e.g. apple cider, white, balsamic, red wine) - Fermented plant-based yogurt or kefir   - I have consumed this food   ▫ 1 time per month or less  ▫ 2-3 times per month  ▫ 1-2 times per week  ▫ 3-4 times per week  ▫ 5-6 times per week  ▫ 1 time per day  ▫ 2-3 times per day  ▫ 4 or more times per day |
| Q19Including foods selected in Q18 only, Multiple choice: single response allowed)  *Each selected food is displayed one at a time with the answer options as a multiple-choice question:* | Please indicate the primary reason that you have consumed each of the fermented foods that you selected in Q18 which will be shown one at a time below. To go to the next food, you can press the small grey arrow button.  ▫ The product tastes good  ▫ Promote overall health  ▫ Manage a disease or condition  ▫ Cultural or heritage/tradition reasons  ▫ Other (please enter): input box |
| Q20  (multiple choice: single response allowed) | Do you consume non-heated (non-pasteurized) fermented foods such as non-heated fermented vegetables (e.g. non-heated kimchi or non-heated sauerkraut) or non-heated sausage (e.g. non-heated salami)? These are typically found in the refrigerated section of a store.   - Yes - No - Not sure |
| Q21  (multiple choice: single response allowed, if yes then free response) | Do you make fermented foods at home?   - Yes [please enter the foods separated by a comma (e.g. yogurt, kimchi)]: - No |
| Q22  (multiple choice: single response allowed, if yes then free response) | If you have children in your household, do they eat fermented foods?   - Yes [please enter the foods separated by a comma (e.g. yogurt, kimchi)]: - No - Not Applicable |
| Q23  (checkbox list: multiple responses allowed, free response for Website, Blog posts, Other) | What type of resources do you use as sources of information on how to ferment foods at home, if any?   - University Extension publications (examples: Gardening at USask, National Center for Home Food Preservation, University of California Cooperative Extension Resources, etc.) - United States Department of Agriculture (USDA) Complete Guide to Home Canning - Textbooks (examples: Microbiology and Technology of Fermented Foods, Fermentation Microbiology and Biotechnology, Principles of Fermentation Technology, etc.) - Other Books (examples: Farmhouse Culture Guide to Fermentation, NOMA guide to Fermentation, the Art of Fermentation, Fermented Vegetables, Wild Fermentation, etc.) - Website (please specify): - Blog posts (please specify): - Other (please specify): - None - I do not ferment foods at home and I do not use resources on how to do so |
| **Block 3: Perceptions Around Fermented Foods** | |
| **Question (Q) number and type of response allowed** | **Question text included in survey** |
| Q24  (multiple choice: single response allowed) | Would you make fermented foods if you knew how to prepare them?   - Yes - No - I already make fermented foods at home - Not sure |
| Q25  (multiple choice: single response allowed) | Please choose the response that best describes the availability of the fermented foods you would like to purchase.   - Easily available - Mostly available - Somewhat difficult to find - Not available - I do not purchase fermented foods |
| Q26  (multiple choice: single response allowed) | Is there a history of fermented foods in your culture?   - Yes - No - Not sure |
| Q27  (multiple choice: single response allowed) | Did older generations in your family make fermented foods? (e.g. your parents, grandparents etc.)   - Yes - No - Not sure |
| Q28  (multiple choice: single response allowed) | Do you like the taste of fermented foods?   - Yes - No - Not sure |
| Q29  (multiple choice: single response allowed, if yes then free response) | Do you think fermented foods are good for you?   - Yes [please specify the primary reason(s)]: - No - Not sure |
| Q30  (multiple choice: single response allowed) | Are you open to trying new fermented foods?   - Yes - No - Not sure |
| Q31  (checkbox list: multiple responses allowed, free response for Other) | What factors least appeal to you about trying new fermented foods?   - Taste - Texture - Smell - Appearance - Fermentation process - Safety - Cost - Other (please specify): |
| Q32  (multiple choice: single response allowed) | Do you perceive probiotics and fermented foods to be the same thing?   - Yes - No - Not sure |
| Q33  (multiple choice: single response allowed) | Microorganisms (e.g. bacteria, yeast) are used to make fermented foods.   - Yes - No - Not sure |
| Q34  (multiple choice: single response allowed) | Fermented foods are completely safe.   - Yes - No - Not sure |
| Q35  (checkbox list: multiple responses allowed, free response for Other) | What educational materials would you prefer to use to access more information about fermented foods?   - Canada’s Food Guide / Canada’s Dietary Guidelines (published by Health Canada) - Expert-developed brochures - Expert-developed website - Expert-developed blog - Expert-developed video(s) - Other (please specify): - I would not prefer to use any educational materials |
| Survey Completion Message | **You have completed this survey!**  Your responses have been recorded and we thank you for your participation. Your time and contributions to this research are appreciated. |
| Ineligible End of Survey Message (Q1 or Q2) | Thank you for your interest in this survey! You were not eligible to be included in the study at this time. We appreciate your time. |

**Supplementary Table 2. Knowledge of fermentation process by demographic characteristics. N(%).**

| **Q33: Microorganisms (e.g. bacteria, yeast) are used to make fermented foods.** | | | | | |
| --- | --- | --- | --- | --- | --- |
| **Characteristics** | Not Sure | No | Yes | Unknown | Chi sq (p-value) |
| **Totals** | 1266 (31.3) | 371 (9.2) | 2396 (59.6) | 12 (0.3) |  |
| **Age** |  |  |  |  |  |
| Mean (standard deviation) | 49.5 (17.4) | 39.9 (16.3) | 47.7 (16.9) | 34.3 (18.4) | (<.0001*) |
| Median (IQR) | 50 (35, 64) | 36 (25, 50) | 47 (33, 62) | 27.5 (23, 35) | 90.581 (<.0001*) |
| 18-25 | 154 (12.2) | 95 (25.6) | 263 (11.0) | 6 (50.0) | 125.82 (<.0001*) |
| 26-35 | 184 (14.5) | 83 (22.4) | 431 (18.0) | 3 (25.0) |  |
| 36-45 | 177 (14.0) | 70 (18.9) | 441 (18.4) | 1 (8.3) |  |
| 46-55 | 212 (16.7) | 56 (15.1) | 405 (16.9) | 0 (0.0) |  |
| 56-65 | 260 (20.5) | 28 (7.5) | 411 (17.2) | 0 (0.0) |  |
| 66+ | 279 (22.0) | 39 (10.5) | 445 (18.6) | 2 (16.7) |  |
| **BMI** |  |  |  |  |  |
| Mean (standard deviation) | 27.3 (5.7) | 26.5 (5.4) | 27.1 (5.3) | 25.6 (4.3) | (.118) |
| median (IQR) | 26.3 (23, 30) | 25.3 (23, 30) | 26.4 (23, 30) | 28.3 (23, 29) | 4.0808 (.1300) |
| Missing | 230 (18.2) | 94 (25.3) | 356 (14.9) | 5 (41.7) |  |
| **Canadian Province** |  |  |  |  | 33.459 (.1492**) |
| Alberta | 152 (12.0) | 45 (12.1) | 306 (12.8) | 1 (8.3) |  |
| British Columbia | 172 (13.6) | 33 (8.9) | 326 (13.6) | 3 (25.0) |  |
| Manitoba | 70 (5.5) | 15 (4.0) | 106 (4.4) | 0 (0.0) |  |
| Newfoundland and Labrador | 34 (2.7) | 6 (1.6) | 52 (2.2) | 0 (0.0) |  |
| New Brunswick | 44 (3.5) | 9 (2.4) | 51 (2.1) | 0 (0.0) |  |
| Nova Scotia | 39 (3.1) | 12 (3.5) | 76 (3.2) | 1 (8.3) |  |
| Northwest Territories | 0 (0.0) | 0 (0.0) | 1 (0.0) | 0 (0.0) |  |
| Nunavut | 0 (0.0) | 0 (0.0) | 1 (0.0) | 0 (0.0) |  |
| Ontario | 562 (44.4) | 172 (46.4) | 1046 (43.7) | 3 (25.0) |  |
| Prince Edward Island | 7 (0.6) | 2 (0.5) | 20 (0.8) | 0 (0.0) |  |
| Quebec | 129 (10.2) | 61 (16.4) | 297 (12.4) | 2 (16.7) |  |
| Saskatchewan | 33 (2.6) | 12 (3.2) | 80 (3.3) | 2 (16.7) |  |
| Yukon | 1 (0.1) | 0 (0.0) | 2 (0.1) | 0 (0.0) |  |
| Outside of Canada | 23 (1.8) | 3 (0.8) | 32 (1.3) | 0 (0.0) |  |
| **Sex** |  |  |  |  | 21.691 (<.0001**) |
| Male | 562 (44.4) | 213 (57.4) | 1151 (48.0) | 7 (58.3) |  |
| Female | 698 (55.1) | 155 (41.8) | 1234 (51.5) | 4 (33.3) |  |
| Other | 5 (0.4) | 3 (0.8) | 9 (0.4) | 1 (8.3) |  |
| Unknown | 1 (0.1) | 0 (0.0) | 2 (0.1) | 0 (0.0) |  |
| **Highest Level of Education** |  |  |  |  | 114.52 (<.0001*) |
| Elementary | 379 (29.9) | 74 (19.9) | 427 (17.8) | 2 (16.7) |  |
| Trade School | 88 (7.0) | 27 (7.3) | 170 (7.1) | 1 (8.3) |  |
| Community College | 353 (27.9) | 87 (23.5) | 563 (23.5) | 1 (8.3) |  |
| University (undergrad) | 306 (24.2) | 119 (32.1) | 841 (35.1) | 4 (33.3) |  |
| University (grad) | 137 (10.8) | 64 (17.3) | 395 (16.5) | 3 (25.0) |  |
| Unknown | 3 (0.2) | 0 (0.0) | 0 (0.0) | 1 (8.3) |  |
| **Income Level** |  |  |  |  | 54.401 (.0001*) |
| < 30,000 | 281 (22.2) | 67 (18.1) | 342 (14.3) | 3 (25.0) |  |
| 30,000-50,000 | 271 (21.4) | 77 (20.8) | 453 (18.9) | 2 (16.7) |  |
| 51,000-80,000 | 304 (24.0) | 99 (26.7) | 622 (26.0) | 3 (25.0) |  |
| 81,000-120,000 | 237 (18.7) | 75 (20.2) | 527 (22.0) | 2 (16.7) |  |
| >120,000 | 170 (13.4) | 53 (14.3) | 448 (18.7) | 1 (8.3) |  |
| Unknown | 3 (0.2) | 0 (0.0) | 4 (0.2) | 1 (8.3) |  |
| **Living Area** |  |  |  |  | 10.688 (0.0303*) |
| Rural | 252 (19.9) | 75 (20.2) | 478 (19.9) | 4 (33.3) |  |
| Suburban | 518 (40.9) | 128 (34.5) | 869 (36.3) | 3 (25.0) |  |
| Urban | 496 (39.2) | 168 (45.3) | 1049 (43.8) | 5 (41.7) |  |
| **Do You Consider Yourself Healthy?** |  |  |  |  | 13.249 (.0101*) |
| Not Sure | 162 (12.8) | 34 (9.2) | 233 (9.7) | 1 (8.3) |  |
| No | 188 (14.8) | 44 (11.9) | 323 (13.5) | 1 (8.3) |  |
| Yes | 915 (72.3) | 293 (79.0) | 1840 (76.8) | 10 (83.3) |  |
| Unknown | 1 (0.1) | 0 (0.0) | 0 (0.0) | 0 (0.0) |  |
| **How many times per year do you go to the doctor?** |  |  |  |  | 7.7576 (.2564) |
| 0 | 175 (13.8) | 65 (17.5) | 344 (14.4) | 2 (16.7) |  |
| 1 | 435 (34.4) | 134 (36.1) | 788 (32.9) | 5 (41.7) |  |
| 2-4. | 517 (40.8) | 139 (37.5) | 1022 (42.7) | 4 (33.3) |  |
| 5 or more | 139 (11.0) | 33 (8.9) | 242 (10.1) | 1 (8.3) |  |
| **How frequently do you consider health benefits when you choose food to eat?** |  |  |  |  | 64.426 (<.0001*) |
| Never | 86 (6.8) | 24 (6.5) | 70 (2.9) | 2 (16.7) |  |
| Sometimes | 608 (48.0) | 190 (51.2) | 1019 (42.5) | 4 (33.3) |  |
| Most of the time | 474 (37.4) | 118 (31.8) | 1054 (44.0) | 5 (41.7) |  |
| Always | 98 (7.7) | 39 (10.5) | 253 (10.6) | 1 (8.3) |  |
| **What dietary pattern best describes the way you eat?** |  |  |  |  | 178.06 (<.0001**) |
| Omnivorous | 1159 (91.5) | 264 (71.2) | 2174 (90.7) | 9 (75.0) |  |
| Pescatarian | 44 (3.5) | 70 (18.9) | 91 (3.8) | 2 (16.7) |  |
| Vegetarian | 45 (3.6) | 25 (6.7) | 105 (4.4) | 0 (0.0) |  |
| Vegan | 13 (1.0) | 6 (1.6) | 22 (0.9) | 0 (0.0) |  |
| Unknown | 5 (0.4) | 6 (1.6) | 4 (0.2) | 1 (8.3) |  |
| **Indicates statistically significant differences between groups, ** Chi-squared approximation unreliable due to low frequencies.* | | | | | |

**Supplementary Table 3. Knowledge of FF definition by demographic characteristics. N(%).**

| **Q32: Do you perceive probiotics and fermented foods to be the same thing?** | | | | | |
| --- | --- | --- | --- | --- | --- |
| **Characteristics** | Not Sure | No | Yes | Unknown | Chi sq (p-value) |
| **Totals** | 1509 (37.3) | 1643 (40.6) | 879 (21.7) | 14 (0.3) |  |
| **Age** |  |  |  |  |  |
| Mean (standard deviation) | 50.5 (17.2) | 46.7 (17.3) | 44.1 (16.3) | 33.9 (14.5) | (<.0001*) |
| Median (IQR) | 52 (36-65) | 46 (32- 62) | 42 (31- 57) | 32 (23, 36) | 84.686 (<.0001*) |
| 18-25 | 158 (10.5) | 235 (14.3) | 120 (13.7) | 5 (35.7) | 103.15 (<.0001*) |
| 26-35 | 212 (14.0) | 289 (17.6) | 195 (22.2) | 5 (35.7) |  |
| 36-45 | 216 (14.3) | 277 (16.9) | 194 (22.1) | 2 (14.3) |  |
| 46-55 | 262 (17.4) | 272 (16.6) | 139 (15.8) | 0 (0.0) |  |
| 56-65 | 306 (20.3) | 270 (16.4) | 122 (13.9) | 1 (7.1) |  |
| 66+ | 355 (23.5) | 300 (18.3) | 109 (12.4) | 1 (7.1) |  |
| **BMI** |  |  |  |  |  |
| Mean (standard deviation) | 27.1 (5.4) | 27.2 (5.4) | 26.9 (5.5) | 27.2 (3.6) | (.6600) |
| Median (IQR) | 26.3 (23- 30) | 26.4 (23- 30) | 25.8 (23- 30) | 28.5 (24, 29) | 0.9206 (.6311) |
| Missing | 221 (14.6) | 276 (16.8) | 183 (20.8) | 5 (35.7) |  |
| **Canadian Province** |  |  |  |  | 39.995 (.0391**) |
| Alberta | 175 (11.6) | 215 (13.1) | 113 (12.9) | 1 (7.1) |  |
| British Columbia | 220 (14.6) | 213 (13.0) | 97 (11.0) | 4 (28.6) |  |
| Manitoba | 75 (5.0) | 63 (3.8) | 53 (6.0) | 0 (0.0) |  |
| Newfoundland & Labrador | 42 (2.8) | 35 (2.1) | 15 (1.7) | 0 (0.0) |  |
| New Brunswick | 45 (3.0) | 44 (2.7) | 15 (1.7) | 0 (0.0) |  |
| Nova Scotia | 55 (3.6) | 53 (3.2) | 21 (2.4) | 0 (0.0) |  |
| Northwest Territories | 0 (0.0) | 0 (0.0) | 1 (0.1) | 0 (0.0) |  |
| Nunavut | 0 (0.0) | 1 (0.1) | 0 (0.0) | 0 (0.0) |  |
| Ontario | 660 (43.7) | 722 (43.9) | 396 (45.1) | 5 (35.7) |  |
| Prince Edward Island | 7 (0.5) | 11 (0.7) | 11 (1.3) | 0 (0.0) |  |
| Quebec | 160 (10.6) | 213 (13.0) | 114 (13.0) | 2 (14.3) |  |
| Saskatchewan | 45 (3.0) | 54 (3.3) | 26 (3.0) | 2 (14.3) |  |
| Yukon | 1 (0.1) | 1 (0.1) | 1 (0.1) | 0 (0.0) |  |
| Outside of Canada | 24 (1.6) | 18 (1.1) | 16 (1.8) | 0 (0.0) |  |
| **Sex** |  |  |  |  | 1.1862 (.8804**) |
| Male | 713 (47.2) | 790 (48.1) | 422 (48.0) | 8 (57.1) |  |
| Female | 787 (52.2) | 846 (51.5) | 453 (51.5) | 5 (35.7) |  |
| Other | 8 (0.5) | 5 (0.3) | 4 (0.5) | 1 (7.1) |  |
| Unknown | 1 (0.1) | 2 (0.1) | 0 (0.0) | 0 (0.0) |  |
| **Highest Level of Education** |  |  |  |  | 51.203 (<.0001*) |
| Elementary | 389 (25.8) | 354 (21.5) | 138 (15.7) | 1 (7.1) |  |
| Trade School | 115 (7.6) | 111 (6.8) | 59 (6.7) | 1 (7.1) |  |
| Community College | 379 (25.1) | 407 (24.8) | 217 (24.7) | 1 (7.1) |  |
| University (undergrad) | 435 (28.8) | 536 (32.6) | 293 (33.3) | 6 (42.9) |  |
| University (grad) | 189 (12.5) | 234 (14.2) | 172 (19.6) | 4 (28.6) |  |
| Unknown | 2 (0.1) | 4 (0.2) | 0 (0.0) | 1 (7.1) |  |
| **Income Level** |  |  |  |  | 23.421 (<.0029*) |
| < 30,000 | 297 (19.7) | 272 (16.6) | 120 (13.7) | 4 (28.6) |  |
| 30,000-50,000 | 315 (20.9) | 323 (19.7) | 163 (18.5) | 2 (14.3) |  |
| 51,000-80,000 | 377 (25.0) | 411 (25.0) | 237 (27.0) | 3 (21.4) |  |
| 81,000-120,000 | 293 (19.4) | 341 (208) | 206 (23.4) | 1 (7.1) |  |
| >120,000 | 225 (14.9) | 292 (17.8) | 152 (17.3) | 3 (21.4) |  |
| Unknown | 2 (0.1) | 4 (0.2) | 1 (0.1) | 1 (7.1) |  |
| **Living Area** |  |  |  |  | 4.977 (0.2897) |
| Rural | 311 (20.6) | 337 (20.5) | 157 (17.9) | 4 (28.6) |  |
| Suburban | 555 (36.8) | 631 (38.4) | 328 (37.3) | 4 (28.6) |  |
| Urban | 643 (42.6) | 675 (41.1) | 394 (44.8) | 6 (42.9) |  |
| **Consider yourself healthy?** |  |  |  |  | 39.946 (<.0001*) |
| Not Sure | 213 (14.1) | 160 (9.7) | 56 (6.4) | 1 (7.1) |  |
| No | 204 (13.5) | 239 (14.5) | 113 (12.9) | 0 (0.0) |  |
| Yes | 1092 (72.4) | 1243 (75.7) | 710 (80.8) | 13 (92.9) |  |
| Unknown | 0 (0.0) | 1 (0.1) | 0 (0.0) | 0 (0.0) |  |
| **How often go to the doctor?** |  |  |  |  | 10.524 (.1043) |
| 0 | 226 (15.0) | 254 (15.5) | 106 (12.1) | 0 (0.0) |  |
| 1 | 496 (32.9) | 572 (34.8) | 288 (32.8) | 6 (42.9) |  |
| 2-4 | 628 (41.6) | 650 (39.6) | 397 (45.2) | 7 (50.0) |  |
| 5 or more | 159 (10.5) | 167 (10.2) | 88 (10.0) | 1 (7.1) |  |
| **Consider health benefits when you choose food to eat?** |  |  |  |  | 51.03 (<.0001*) |
| Never | 82 (5.4) | 68 (4.1) | 30 (3.4) | 2 (14.3) |  |
| Sometimes | 700 (46.4) | 792 (48.2) | 325 (37.0) | 4 (28.6) |  |
| Most of the time | 603 (40.0) | 639 (38.9) | 401 (45.6) | 8 (57.1) |  |
| Always | 124 (8.2) | 144 (8.8) | 123 (14.0) | 0 (0.0) |  |
| **Dietary patterns** |  |  |  |  | 59.246 (<.0001*) |
| Omnivorous | 1402 (92.9) | 1462 (89.0) | 731 (83.2) | 11 (78.6) |  |
| Pescatarian | 46 (3.0) | 81 (4.9) | 78 (8.9) | 2 (14.3) |  |
| Vegetarian | 46 (0.3) | 73 (4.4) | 56 (6.4) | 0 (0.0) |  |
| Vegan | 11 (0.7) | 20 (1.2) | 9 (1.0) | 1 (7.1) |  |
| Unknown | 4 (0.3) | 7 (0.4) | 5 (0.6) | 0 (0.0) |  |
| **Indicates statistically significant differences between groups, ** Chi-squared approximation unreliable due to low frequencies.* | | | | | |

**Supplementary Table 4. Perceived safety and knowledge of fermented foods by demographic characteristics. N(%).**

| **Q34: Fermented foods are completely safe.** | | | | | |
| --- | --- | --- | --- | --- | --- |
| Characteristics | Not Sure | No | Yes | Unknown | Chi sq (p-value) |
| **Totals** | 1823 (45.1) | 472 (11.7) | 1737 (42.9) | 13 (0.3) |  |
| **Age** |  |  |  |  |  |
| Mean (standard deviation) | 48.7 (17.5) | 40.3 (15.9) | 48.2 (16.8) | 34.0 (17.6) | (<.0001*) |
| Median (IQR) | 49 (34-64) | 37.5 (27- 52) | 48 (34-63) | 30 (23, 34) | 93.705 (<.0001*) |
| 18-25 | 229 (12.6) | 112 (23.7) | 171 (9.8) | 6 (46.2) | 115.73 (<.0001**) |
| 26-35 | 283 (15.5) | 104 (22.0) | 310 (17.8) | 4 (30.8) |  |
| 36-45 | 281 (15.4) | 88 (18.6) | 319 (18.4) | 1 (7.7) |  |
| 46-55 | 303 (16.6) | 70 (14.8) | 300 (17.3) | 0 (0.0) |  |
| 56-65 | 335 (18.4) | 60 (12.7) | 304 (17.5) | 0 (0.0) |  |
| 66+ | 392 (21.5) | 38 (8.1) | 333 (19.2) | 2 (15.4) |  |
| **BMI** |  |  |  |  |  |
| Mean (standard deviation) | 27.3 (5.7) | 26.6 (5.5) | 26.9 (5.1) | 25.6 (4.3) | (.029*) |
| Median (IQR) | 26.4 (23- 31) | 25.4 (23- 30) | 26.3 (23- 30) | 28.3 (23, 29) | 6.1924 (.0452*) |
| Missing | 297 (16.3) | 113 (23.9) | 269 (15.5) | 6 (46.2) |  |
| **Canadian Province** |  |  |  |  | 44.617 (.01296**) |
| Alberta | 207 (11.4) | 60 (12.7) | 236 (13.6) | 1 (7.7) |  |
| British Columbia | 237 (13.0) | 53 (11.2) | 240 (13.8) | 4 (30.8) |  |
| Manitoba | 86 (4.7) | 18 (3.8) | 87 (5.0) | 0 (0.0) |  |
| Newfoundland & Labrador | 52 (2.9) | 3 (0.6) | 37 (2.1) | 0 (0.0) |  |
| New Brunswick | 52 (2.9) | 8 (1.7) | 44 (2.5) | 0 (0.0) |  |
| Nova Scotia | 60 (3.3) | 15 (3.2) | 53 (3.1) | 1 (7.7) |  |
| Northwest Territories | 0 (0.0) | 0 (0.0) | 1 (0.1) | 0 (0.0) |  |
| Nunavut | 0 (0.0) | 0 (0.0) | 1 (0.1) | 0 (0.0) |  |
| Ontario | 810 (44.4) | 211 (44.7) | 759 (43.7) | 3 (23.1) |  |
| Prince Edward Island | 15 (0.8) | 6 (1.3) | 8 (0.5) | 0 (0.0) |  |
| Quebec | 226 (12.4) | 80 (16.9) | 181 (10.4) | 2 (15.4) |  |
| Saskatchewan | 53 (2.9) | 8 (1.7) | 64 (3.7) | 2 (15.4) |  |
| Yukon | 1 (0.1) | 1 (0.2) | 1 (0.1) | 0 (0.0) |  |
| Outside of Canada | 24 (1.3) | 9 (1.9) | 25 (1.4) | 0 (0.0) |  |
| **Sex** |  |  |  |  | 11.159 (<.02483**) |
| Male | 855 (46.9) | 254 (53.8) | 816 (47.0) | 8 (61.5) |  |
| Female | 959 (52.6) | 214 (45.3) | 914 (52.6) | 4 (30.8) |  |
| Other | 8 (0.4) | 4 (0.8) | 5 (0.3) | 1 (7.7) |  |
| Unknown | 1 (0.1) | 0 (0.0) | 2 (0.1) | 0 (0.0) |  |
| **Highest Level of Education** |  |  |  |  | 50.049 (<.0001*) |
| Elementary | 461 (25.3) | 80 (16.9) | 339 (19.5) | 2 (15.4) |  |
| Trade School | 127 (7.0) | 36 (7.6) | 121 (7.0) | 2 (15.4) |  |
| Community College | 490 (26.9) | 107 (22.7) | 406 (23.4) | 1 (7.7) |  |
| University (undergrad) | 522 (28.6) | 162 (34.3) | 582 (33.5) | 4 (30.8) |  |
| University (grad) | 221 (12.1) | 87 (18.4) | 288 (16.6) | 3 (23.1) |  |
| Unknown | 2 (0.1) | 0 (0.0) | 1 (0.1) | 1 (7.7) |  |
| **Income Level** |  |  |  |  | 29.008 (<.0001*) |
| < 30,000 | 361 (19.8) | 78 (16.5) | 251 (14.5) | 3 (23.1) |  |
| 30,000-50,000 | 378 (20.7) | 89 (18.9) | 334 (19.2) | 2 (15.4) |  |
| 51,000-80,000 | 456 (25.0) | 129 (27.3) | 440 (25.3) | 3 (23.1) |  |
| 81,000-120,000 | 339 (18.6) | 104 (22.0) | 395 (22.7) | 3 (23.1) |  |
| >120,000 | 285 (15.6) | 71 (15.0) | 315 (18.1) | 1 (7.7) |  |
| Unknown | 4 (0.2) | 1 (0.2) | 2 (0.1) | 1 (7.7) |  |
| **Living Area** |  |  |  |  | 9.6488 (.04678*) |
| Rural | 375 (20.6) | 90 (19.1) | 340 (19.6) | 4 (30.8) |  |
| Suburban | 720 (39.5) | 178 (37.7) | 617 (35.5) | 3 (23.1) |  |
| Urban | 728 (39.9) | 204 (43.2) | 780 (44.9) | 6 (46.2) |  |
| **Consider yourself healthy?** |  |  |  |  | 48.236 (<.0001*) |
| Not Sure | 256 (14.0) | 38 (8.1) | 135 (7.8) | 1 (7.7) |  |
| No | 267 (14.6) | 71 (15.0) | 217 (12.5) | 1 (1.1) |  |
| Yes | 1300 (71.3) | 363 (76.9) | 1384 (79.7) | 11 (84.6) |  |
| Unknown | 0 (0.0) | 0 (0.0) | 1 (0.0) | 0 (0.0) |  |
| **How often go to the doctor?** |  |  |  |  | 7.3253 (.2918) |
| 0 | 266 (14.6) | 75 (15.9) | 243 (14.0) | 2 (15.4) |  |
| 1 | 626 (34.3) | 164 (34.7) | 567 (32.6) | 5 (38.5) |  |
| 2-4 | 743 (40.8) | 177 (37.5) | 757 (43.6) | 5 (38.5) |  |
| 5 or more | 188 (10.3) | 56 (11.9) | 170 (9.8) | 1 (7.7) |  |
| **Consider health benefits when you choose food to eat?** |  |  |  |  | 76.753 (<.0001*) |
| Never | 107 (5.9) | 25 (5.3) | 48 (2.8) | 2 (15.4) |  |
| Sometimes | 896 (49.1) | 225 (47.7) | 695 (40.0) | 5 (38.5) |  |
| Most of the time | 693 (38.0) | 175 (37.1) | 778 (44.8) | 5 (38.5) |  |
| Always | 127 (7.0) | 47 (10.0) | 216 (12.4) | 1 (7.7) |  |
| **Dietary patterns** |  |  |  |  | 60.085 (<.0001**) |
| Omnivorous | 1671 (91.7) | 378 (80.1) | 1548 (89.1) | 9 (69.2) |  |
| Pescatarian | 65 (3.6) | 53 (11.2) | 86 (5.0) | 3 (23.1) |  |
| Vegetarian | 65 (3.6) | 28 (5.9) | 82 (4.7) | 0 (0.0) |  |
| Vegan | 14 (0.8) | 9 (1.9) | 18 (1.0) | 0 (0.0) |  |
| Unknown | 8 (0.4) | 4 (0.8) | 3 (0.2) | 1 (7.7) |  |

**Indicates statistically significant differences between groups, ** Chi-squared approximation unreliable due to low frequencies.*

**Supplementary Table 5.** Distribution of responses towards making fermented foods at home by demographic factors. N(%).

| **Q21: Do you make fermented foods at home? N (%)** | | | | |
| --- | --- | --- | --- | --- |
| **Characteristics** | **No** | **Yes** | **Unknown** | **Chi sq (p-value)** |
| **Totals** | 3663 (90.6) | 366 (9.0) | 16 (0.4) |  |
| **Age** |  |  |  |  |
| Mean (standard deviation) | 48.0 (17.3) | 43.5 (16.5) | 34.9 (15.8) | (<.0001*) |
| Median (IQR) | 48 (34-63) | 42 (30-57) | 27.5 (23- 42) | 22.558 (<.0001*) |
| 18-25 | 454 (12.4) | 56 (15.3) | 8 (50.0) | 27.857 (<.0001*) |
| 26-35 | 607 (16.6) | 93 (25.4) | 1 (6.2) |  |
| 36-45 | 623 (17.0) | 62 (16.9) | 4 (25.0) |  |
| 46-55 | 615 (16.8) | 57 (15.6) | 1 (6.2) |  |
| 56-65 | 648 (17.7) | 50 (13.7) | 1 (6.2) |  |
| 66+ | 716 (19.5) | 48 (13.1) | 1 (6.2) |  |
| **BMI** |  |  |  |  |
| Mean (standard deviation) | 27.2 (5.4) | 26.4 (5.5) | 29.7 (6.0) | (.0181*) |
| Median (IQR) | 26.4 (23- 30) | 25.3 (22-29) | 29.3 (26-35) | 8.8663 (.0029*) |
| Missing | 612 (16.7) | 68 (18.6) | 5 (31.2) |  |
| **Canadian Province** |  |  |  | 29.787 (.0051**) |
| Alberta | 458 (12.5) | 45 (12.3) | 1 (6.2) |  |
| British Columbia | 480 (13.1) | 49 (13.4) | 5 (31.2) |  |
| Manitoba | 179 (4.9) | 11 (3.0) | 1 (6.2) |  |
| Newfoundland and Labrador | 83 (2.3) | 7 (1.9) | 2 (12.5) |  |
| New Brunswick | 101 (2.8) | 3 (0.8) | 0 (0.0) |  |
| Nova Scotia | 116 (3.2) | 13 (3.6) | 0 (0.0) |  |
| Northwest Territories | 0 (0.0) | 1 (0.3) | 0 (0.0) |  |
| Nunavut | 1 (0.0) | 0 (0.0) | 0 (0.0) |  |
| Ontario | 1605 (43.8) | 173 (47.3) | 5 (31.2) |  |
| Prince Edward Island | 25 (0.7) | 3 (0.8) | 1 (6.2) |  |
| Quebec | 453 (12.4) | 36 (9.8) | 0 (0.0) |  |
| Saskatchewan | 113 (3.1) | 13 (3.6) | 1 (6.2) |  |
| Yukon | 2 (0.1) | 1 (0.3) | 0 (0.0) |  |
| Outside of Canada | 47 (1.3) | 11 (3.0) | 0 (0.0) |  |
| **Sex** |  |  |  | 5.679 (.0585**) |
| Male | 1771 (48.3) | 154 (42.1) | 8 (50.0) |  |
| Female | 1873 (51.1) | 211 (57.7) | 7 (43.8) |  |
| Other | 16 (0.4) | 1 (0.3) | 1 (6.2) |  |
| Unknown | 3 (0.1) | 0 (0.0) | 0 (0.0) |  |
| **Highest Level of Education** |  |  |  | 66.789 (<.0001*) |
| Elementary | 832 (22.7) | 46 (12.6) | 4 (25.0) |  |
| Trade School | 264 (7.2) | 21 (5.7) | 1 (6.2) |  |
| Community College | 939 (25.6) | 63 (17.2) | 2 (12.5) |  |
| University (undergrad) | 1124 (30.7) | 141 (38.5) | 5 (31.2) |  |
| University (grad) | 500 (13.7) | 95 (26.0) | 4 (25.0) |  |
| Unknown | 4 (0.1) | 0 (0.0) | 0 (0.0) |  |
| **Income Level** |  |  |  | 7.9445 (.0936) |
| < 30,000 | 639 (17.4) | 51 (13.9) | 3 (18.8) |  |
| 30,000-50,000 | 733 (20.0) | 62 (16.9) | 8 (50.0) |  |
| 51,000-80,000 | 929 (25.4) | 96 (26.2) | 3 (18.8) |  |
| 81,000-120,000 | 747 (20.4) | 92 (25.1) | 2 (12.5) |  |
| >120,000 | 607 (16.6) | 65 (17.8) | 0 (0.0) |  |
| Unknown | 8 (0.2) | 0 (0.0) | 0 (0.0) |  |
| **Living Area** |  |  |  | 0.6951 (0.7064) |
| Rural | 733 (20.0) | 71 (19.4) | 5 (31.2) |  |
| Suburban | 1381 (37.7) | 132 (36.1) | 5 (31.2) |  |
| Urban | 1549 (42.3) | 163 (44.5) | 6 (37.5) |  |
| **Do You Consider Yourself Healthy?** |  |  |  | 11.487 (.0032*) |
| Not Sure | 401 (10.9) | 25 (6.8) | 4 (25.0) |  |
| No | 518 (14.1) | 38 (10.4) | 0 (0.0) |  |
| Yes | 2743 (74.9) | 303 (82.8) | 12 (75.0) |  |
| Unknown | 1 (0.0) | 0 (0.0) | 0 (0.0) |  |
| **How many times per year do you go to the doctor?** |  |  |  | 6.5331 (.0884) |
| 0 | 536 (14.6) | 47 (12.8) | 3 (18.8) |  |
| 1 | 1242 (33.9) | 117 (32.0) | 3 (18.8) |  |
| 2-4. | 1524 (41.6) | 151 (41.3) | 7 (43.8) |  |
| 5 or more | 361 (9.9) | 51 (13.9) | 3 (18.8) |  |
| **How frequently do you consider health benefits when you choose food to eat?** |  | |  | 53.005 (<.0001*) |
| Never | 171 (4.7) | 9 (2.5) | 2 (12.5) |  |
| Sometimes | 1697 (46.3) | 120 (32.8) | 4 (25.0) |  |
| Most of the time | 1473 (40.2) | 169 (46.2) | 9 (56.2) |  |
| Always | 322 (8.8) | 68 (18.6) | 1 (6.2) |  |
| **What dietary pattern best describes the way you eat?** |  |  |  | 8.4117 (<.0382**) |
| Omnivorous | 3280 (89.5) | 318 (86.9) | 8 (50.0) |  |
| Pescatarian | 186 (5.1) | 17 (4.6) | 4 (25.0) |  |
| Vegetarian | 146 (4.0) | 26 (7.1) | 3 (18.8) |  |
| Vegan | 36 (1.0) | 5 (1.4) | 0 (0.0) |  |
| Unknown | 15 (0.4) | 0 (0.0 | 1 (6.2) |  |
| **Indicates statistically significant differences between groups, ** Chi-squared approximation unreliable due to low frequencies.* | | | | |

**Supplementary Table 6.** Willingness to try making fermented foods at home by demographic characteristics. N(%).

| Q24: Would you make fermented foods if you knew how to prepare them? | | | | | | | |
| --- | --- | --- | --- | --- | --- | --- | --- |
| Characteristics | | **Not Sure** | **No** | **Yes** | **Already make FFs** | **Unknown** | **Chi sq (p-value)** |
| Totals | | 1178 (29.1) | 1459 (36.1) | 1201 (29.7) | 202 (5.0) | 5 (0.1) |  |
| Age | |  |  |  |  |  |  |
| Mean (standard deviation) | | 50.5 (17.2) | 49.9 (17.3) | 42.2 (15.8) |  | 26.4 (6.7) | (<.0001*) |
| Median (IQR) | | 52 (36-65) | 51 (36-64) | 39 (29-54) | 44.5 (17.4) | 23 (23-33) | 189.75  (<.0001*) |
| 18-25 | | 116 (9.8) | 163 (11.2) | 197 (16.4) | 43 (28-59) | 3 (60.) | 209.72  (<.0001**) |
| 26-35 | | 172 (14.6) | 200 (13.7) | 291 (24.2) | 39 (19.3) | 2 (40.0) |  |
| 36-45 | | 166 (14.1) | 236 (16.2) | 250 (20.8) | 36 (17.8) | 0 (0.0) |  |
| 46-55 | | 210 (17.8) | 248 (17.0) | 184 (15.3) | 37 (18.3) | 0 (0.0) |  |
| 56-65 | | 229 (19.4) | 288 (19.7) | 156 (13.0) | 31 (15.3) | 0 (0.0) |  |
| 66+ | | 285 (24.2) | 324 (22.2) | 123 (10.2) | 26 (12.9) | 0 (0.0) |  |
| BMI | |  |  |  |  |  |  |
| Mean (standard deviation) | | 27.5 (5.5) | 27.3 (5.5) | 26.5 (5.2) | 26.7 (5.1) | 26.4 (4.1) | (0.0003*) |
| Median (IQR) | | 26.6 (24-31) | 26.6 (23-30) | 25.6 (23-30) | 25.7 (23-29) | 26.4 (25-28) | 22.651  (0.00015*) |
| Missing | |  |  |  |  |  |  |
| Canadian Province | |  |  |  |  |  | 96.429  (0.00018**) |
| Alberta | | 135 (11.5) | 179 (12.3) | 161 (13.4) | 28 (13.9) | 1 (20.0) |  |
| British Columbia | | 173 (14.7) | 186 (12.7) | 148 (12.3) | 27 (13.4) | 0 (0.0) |  |
| Manitoba | | 64 (5.4) | 70 (4.8) | 52 (4.3) | 5 (2.5) | 0 (0.0) |  |
| Newfoundland and Labrador | | 41 (3.5) | 27 (1.9) | 22 (1.8) | 2 (1.0) | 0 (0.0) |  |
| New Brunswick | | 34 (2.9) | 31 (2.1) | 37 (3.1) | 2 (1.0) | 0 (0.0) |  |
| Nova Scotia | | 44 (3.7) | 46 (3.2) | 35 (2.9) | 4 (2.0) | 0 (0.0) |  |
| Northwest Territories | | 0 (0.0) | 0 (0.0) | 1 (0.1) | 0 (0.0) | 0 (0.0) |  |
| Nunavut | | 0 (0.0) | 1 (0.1) | 0 (0.0) | 0 (0.0) | 0 (0.0) |  |
| Ontario | | 481 (40.8) | 659 (45.2) | 551 (45.9) | 90 (44.6) | 2 (40.0) |  |
| Prince Edward Island | | 7 (0.6) | 11 (0.8) | 11 (0.9) | 0 (0.0) | 0 (0.0) |  |
| Quebec | | 147 (12.5) | 181 (12.4) | 133 (11.1) | 28 (13.9) | 0 (0.0) |  |
| Saskatchewan | | 39 (3.3) | 45 (3.1) | 34 (2.8) | 7 (3.5) | 2 (40.0) |  |
| Yukon | | 1 (0.1) | 0 (0.0) | 0 (0.0) | 2 (1.0) | 0 (0.0) |  |
| Outside of Canada | | 12 (1.0) | 23 (1.6) | 16 (1.3) | 7 (3.5) | 0 (0.0) |  |
| Sex | |  |  |  |  |  | 29.765  (0.003**) |
| Male | | 508 (43.1) | 715 (49.0) | 624 (52.0) | 82 (40.6) | 4 (80.0) |  |
| Female | | 665(56.5) | 737 (50.5) | 570 (47.5) | 118 (58.4) | 1 (20.0) |  |
| Other | | 5 (0.4) | 6 (0.4) | 5 (0.4) | 2 (1.0) | 0 (0.0) |  |
| Unknown | | 0 (0.0) | 1 (0.1) | 2 (0.2) | 0 (0.0) | 0 (0.0) |  |
| Highest Level of Education | |  |  |  |  |  | 86.315 (<.0001**) |
| Elementary | | 303 (25.7) | 353 (24.2) | 194 (16.2) | 32 (15.8) | 0 (0.0) |  |
| Trade School | | 82 (7.0) | 102 (7.0) | 87 (7.2) | 14 (6.9) | 1 (20.0) |  |
| Community College | | 310 (26.3) | 376 (25.8) | 269 (22.4) | 49 (24.3) | 0 (0.0) |  |
| University(undergrad) | | 339 (28.8) | 440 (30.2) | 419 (34.9) | 68 (33.7) | 4 (80.0) |  |
| University (grad) | | 143 (12.1) | 185 (12.7) | 232 (19.3) | 39 (19.3) | 0 (0.0) |  |
| Unknown | | 1 (0.1) | 3 (0.2) | 0 (0.0) | 0 (0.0) | 0 (0.0) |  |
| Income Level | |  |  |  |  |  | 16.689 (0.673**) |
| < 30,000 | | 215 (18.3) | 258 (17.7) | 190 (15.8) | 29 (14.4) | 1 (20.0) |  |
| 30,000-50,000 | | 247 (21.0) | 281 (19.3) | 236 (19.7) | 37 (18.3) | 2 (40.0) |  |
| 51,000-80,000 | | 293 (24.9) | 378 (25.9) | 299 (24.9) | 56 (27.7) | 2 (40.0) |  |
| 81,000-120,000 | | 224 (19.0) | 292 (20.0) | 277 (23.1) | 48 (23.8) | 0 (0.0) |  |
| >120,000 | | 198 (16.8) | 246 (16.9) | 196 (16.3) | 32 (15.8) | 0 (0.0) |  |
| Unknown | | 1 (0.1) | 4 (0.3) | 3 (0.2) | 0 (0.0) | 0 (0.0) |  |
| Living Area | |  |  |  |  |  | 5.6919 (0.6817**) |
| Rural | | 253 (21.5) | 278 (19.1) | 235 (19.6) | 42 (20.8) | 1 (20.0) |  |
| Suburban | | 431 (36.6) | 576 (39.5) | 436 (36.3) | 73 (36.1) | 2 (40.0) |  |
| Urban | | 494 (41.9) | 605 (41.5) | 530 (44.1) | 87 (43.1) | 2 (40.0) |  |
| Do You Consider Yourself Healthy | |  |  |  |  |  | 42.66 (<.0001**) |
| Not Sure | | 166 (14.1) | 148 (10.1) | 99 (8.2) | 16 (7.9) | 1 (20.0) |  |
| No | | 172 (14.6) | 223 (15.3) | 131 (10.9) | 30 (14.9) | 0 (0.0) |  |
| Yes | | 840 (71.3) | 1088 (74.6) | 970 (80.8) | 156 (77.2) | 4 (80.0) |  |
| Unknown | | 0 (0.0) | 0 (0.0) | 1 (0.1) | 0 (0.0) | 0 (0.0) |  |
| How many times per year do you go to the doctor? | |  |  |  |  |  | 13.521 (0.3323) |
| 0 | | 178 (15.1) | 214 (14.7) | 166 (13.8) | 28 (13.9) | 0 (0.0) |  |
| 1 | | 374 (31.7) | 522 (35.8) | 400 (33.3) | 64 (31.7) | 2 (40.0) |  |
| 2-4. | | 520 (44.1) | 573 (39.3) | 504 (42.0) | 83 (41.1) | 2 (40.0) |  |
| 5 or more | | 106 (9.0) | 150 (10.3) | 131(10.9) | 27 (13.4) | 1 (20.0) |  |
| How frequently do you consider health benefits when you choose food to eat? | |  |  |  |  |  | 141.8 (<.0001**) |
| Never | | 50 (4.2) | 88 (6.0) | 36 (3.0) | 6 (3.0) | 2 (40.0) |  |
| Sometimes | | 537 (45.6) | 753 (51.6) | 458 (38.1) | 72 (35.6) | 1 (20.0) |  |
| Most of the time | | 512 (43.5) | 516 (35.4) | 533 (44.4) | 88 (43.6) | 2 (40.0) |  |
| Always | | 79 (6.7) | 102 (7.0) | 174 (14.5) | 36 (17.8) | 0 (0.0) |  |
| What dietary pattern best describes the way you eat? | |  |  |  |  |  | 54.896 (<.0001**) |
| Omnivorous | | 1103 (93.6) | 1296 (88.8) | 1037 (86.3) | 165 (81.7) | 5 (100.0) |  |
| Pescatarian | | 31 (2.6) | 87 (6.0) | 76 (6.3) | 13 (6.4) | 0 (0.0) |  |
| Vegetarian | | 31 (2.6) | 57 (3.9) | 69 (5.7) | 18 (8.9) | 0 (0.0) |  |
| Vegan | | 10 (0.8) | 13 (0.9) | 14 (1.2) | 4 (2.0) | 0 (0.0) |  |
| Unknown | | 3 (0.3) | 6 (0.4) | 5 (0.4) | 2 (1.0) | 0 (0.0) |  |
|  | **Indicates statistically significant differences between groups, ** Chi-squared approximation unreliable due to low frequencies.* | | | | | | |

**Supplementary Table 7.** Family history of making FFs at home by demographic characteristics. N (%).

| Q27: Did older generations in your family make fermented foods? | | | | | |
| --- | --- | --- | --- | --- | --- |
| Characteristics | **Not Sure** | **No** | **Yes** | **Unknown** | **Chi sq (p-value)** |
| Totals | 979 (24.2) | 1724 (42.6) | 1334 (33.0) | 8 (0.2) |  |
| Age |  |  |  |  |  |
| Mean (standard deviation) | 48.0 (17.1) | 49.6 (17.5) | 44.5 (16.6) | 27.5 (5.9) | (<.0001*) |
| Median (IQR) | 49 (34, 62) | 50 (35, 65) | 42 (31, 58) | 27 (23, 33) | 65.267 (<.0001*) |
| 18-25 | 132 (13.5) | 193 (11.2) | 189 (14.2) | 4 (50.0) | 85.64 (<.0001*) |
| 26-35 | 150 (15.3) | 255 (14.8) | 292 (21.9) | 4 (50.0) |  |
| 36-45 | 160 (16.3) | 278 (16.1) | 251 (18.8) | 0 (0.0) |  |
| 46-55 | 156 (15.9) | 291 (16.9) | 226 (16.9) | 0 (0.0) |  |
| 56-65 | 204 (20.8) | 303 (17.6) | 192 (14.4) | 0 (0.0) |  |
| 66+ | 177 (18.1) | 404 (23.4) | 184 (13.8) | 0 (0.0) |  |
| BMI |  |  |  |  |  |
| Mean (standard deviation) | 27.1 (5.3) | 27.3 (5.5) | 26.8 (5.4) | 27.3 (3.2) | (0.429*) |
| Median (IQR) | 26.3 (23, 30) | 26.3 (23, 31) | 25.9 (23, 30) | 29.0 (26, 29) | 5.7387 (.0567) |
| Missing | 174 (17.8) | 284 (16.5) | 222 (16.6) | 5 (62.5) |  |
| Canadian Province |  |  |  |  | 60.646 (.0001**) |
| Alberta | 123 (12.6) | 186 (10.8) | 194 (14.5) | 1 (12.5) |  |
| British Columbia | 130 (13.3) | 238 (13.8) | 164 (12.3) | 2 (25.0) |  |
| Manitoba | 47 (4.8) | 71 (4.1) | 73 (5.5) | 0 (0.0) |  |
| Newfoundland and Labrador | 26 (2.7) | 40 (2.3) | 26 (1.9) | 0 (0.0) |  |
| New Brunswick | 28 (2.9) | 50 (2.9) | 26 (1.9) | 0 (0.0) |  |
| Nova Scotia | 38 (3.9) | 44 (2.6) | 47 (3.5) | 0 (0.0) |  |
| Northwest Territories | 0 (0.0) | 0 (0.0) | 1 (0.1) | 0 (0.0) |  |
| Nunavut | 0 (0.0) | 0 (0.0) | 1 (0.1) | 0 (0.0) |  |
| Ontario | 418 (42.7) | 761 (44.1) | 602 (45.1) | 2 (25.0) |  |
| Prince Edward Island | 10 (1.0) | 10 (0.6) | 9 (0.7) | 0 (0.0) |  |
| Quebec | 110 (11.2) | 260 (15.1) | 118 (8.8) | 1 (12.5) |  |
| Saskatchewan | 33 (3.4) | 41 (2.4) | 51 (3.8) | 2 (25.0) |  |
| Yukon | 1 (0.1) | 2 (0.1) | 0 (0.0) | 0 (0.0) |  |
| Outside of Canada | 10 (1.0) | 21 (1.2) | 22 (1.6) | 0 (0.0) |  |
| Sex |  |  |  |  | 8.9031 (.0636**) |
| Male | 451 (46.1) | 812 (47.1) | 665 (49.9) | 5 (62.5) |  |
| Female | 520 (53.1) | 907 (52.6) | 662 (49.7) | 2 (25.0) |  |
| Other | 8 (0.8) | 4 (0.2) | 5 (0.4) | 1 (12.5) |  |
| Unknown | 0 (0.) | 1 (0.1) | 2 (0.1) | 0 (0.0) |  |
| Highest Level of Education |  |  |  |  | 95.042 (<.0001*) |
| Elementary | 281 (28.4) | 373 (21.6) | 228 (17.1) | 0 (0.0) |  |
| Trade School | 78 (8.0) | 120 (7.0) | 87 (6.5) | 1 (12.5) |  |
| Community College | 262 (26.8) | 443 (25.7) | 300 (22.5) | 0 (0.0) |  |
| University(undergrad) | 260 (26.6) | 553 (32.1) | 452 (33.9) | 5 (62.5) |  |
| University (grad) | 95 (9.7) | 235 (13.6) | 267 (20.0) | 2 (25.0) |  |
| Unknown | 3 (0.3) | 1 (0.1) | 0 (0.0) | 0 (0.0) |  |
| Income Level |  |  |  |  | 26.764 (.0008*) |
| < 30,000 | 203 (20.7) | 281 (16.3) | 208 (15.6) | 1 (12.5) |  |
| 30,000-50,000 | 221 (22.6) | 332 (19.3) | 248 (18.6) | 2 (25.0) |  |
| 51,000-80,000 | 237 (24.2) | 442 (25.6) | 352 (26.4) | 2 (25.0) |  |
| 81,000-120,000 | 176 (18.0) | 362 (21.0) | 302 (22.6) | 1 (12.5) |  |
| >120,000 | 139 (14.2) | 307 (17.8) | 224 (16.8) | 2 (25.0) |  |
| Unknown | 3 (0.3) | 2 (0.1) | 3 (0.2) | 0 (0.0) |  |
| Living Area |  |  |  |  | 16.33 (.0026*) |
| Rural | 227 (23.2) | 338 (19.6) | 243 (18.2) | 1 (12.5) |  |
| Suburban | 363 (37.1) | 676 (39.2) | 476 (35.7) | 3 (37.5) |  |
| Urban | 389 (39.7) | 710 (41.2) | 615 (46.1) | 4 (50.0) |  |
| Do You Consider Yourself Healthy |  |  |  |  | 10.782 (.0291*) |
| Not Sure | 127 (13.0) | 164 (9.5) | 138 (10.4) | 1 (12.5) |  |
| No | 146 (14.9) | 237 (13.7) | 173 (13.0) | 0 (0.0) |  |
| Yes | 706 (72.1) | 1323 (76.7) | 1022 (76.7) | 7 (87.5) |  |
| Unknown | 0 (0.0) | 0 (0.0) | 1 (0.1) | 0 (0.0) |  |
| How many times per year do you go to the doctor? |  |  |  |  | 11.169 (.0833) |
| 0 | 145 (14.8) | 256 (14.8) | 185 (13.9) | 0 (0.0) |  |
| 1 | 325 (33.2) | 618 (35.8) | 416 (31.2) | 3 (37.5) |  |
| 2-4. | 401 (41.0) | 689 (40.0) | 588 (44.1) | 4 (50.0) |  |
| 5 or more | 108 (11.0) | 161 (9.3) | 145 (10.9) | 1 (12.5) |  |
| How frequently do you consider health benefits when you choose food to eat? |  |  |  |  | 51.114 (<.0001*) |
| Never | 57 (5.8) | 95 (5.5) | 28 (2.1) | 2 (25.0) |  |
| Sometimes | 470 (48.0) | 780 (45.2) | 570 (42.7) | 1 (12.5) |  |
| Most of the time | 376 (38.4) | 705 (40.9) | 565 (42.4) | 5 (67.5) |  |
| Always | 76 (7.8) | 144 (8.4) | 171 (12.8) | 0 (0.0) |  |
| What dietary pattern best describes the way you eat? |  |  |  |  | 42.834 (<.0001*) |
| Omnivorous | 917 (92.0) | 1527 (89.0) | 1155 (86.8) | 7 (87.5) |  |
| Pescatarian | 30 (3.1) | 105 (6.1) | 71 (5.3) | 1 (12.5) |  |
| Vegetarian | 22 (2.2) | 65 (3.8) | 88 (6.6) | 0 (0.0) |  |
| Vegan | 7 (0.7) | 18 (1.0) | 16 (1.2) | 0 (0.0) |  |
| Unknown | 3 (0.3) | 9 (0.5) | 4 (0.3) | 0 (0.0) |  |
| **Indicates statistically significant differences between groups, ** Chi-squared approximation unreliable due to low frequencies.* | | | | | |

**Supplementary Table 8.** Openness to trying fermented foods by demographic characteristics. N(%).

| Q30: Are you open to trying fermented foods? | | | | | |
| --- | --- | --- | --- | --- | --- |
| Characteristics | **Not Sure** | **No** | **Yes** | **Unknown** | **Chi sq (p-value)** |
| Totals | 843 (20.8) | 615 (15.2) | 2578 (63.7) | 9 (0.2) |  |
| Age |  |  |  |  |  |
| Mean (standard deviation) | 52.3 (17.6) | 47.2 (18.7) | 46.1 (16.5) | 25.1 (5.8) | (<.0001*) |
| Median (IQR) | 55 (38- 66) | 47 (31-63) | 45 (33-60) | 23 (21-30) | 81.847 (<.0001*) |
| 18-25 | 94 (11.2) | 105 (17.1) | 313 (12.1) | 6 (66.7) | 130.46 (<.0001*) |
| 26-35 | 95 (11.3) | 101 (16.4) | 502 (19.5) | 3 (333) |  |
| 36-45 | 99 (11.7) | 93 (15.1) | 497 (19.3) | 0 (0.0) |  |
| 46-55 | 135 (16.0) | 84 (13.7) | 454 (17.6) | 0 (0.0) |  |
| 56-65 | 187 (22.2) | 100 (16.3) | 412 (16.0) | 0 (0.0) |  |
| 66+ | 233 (27.6) | 132 (21.5) | 400 (15.5) | 0 (0.0) |  |
| BMI |  |  |  |  |  |
| Mean (standard deviation) | 27.5 (5.6) | 27.3 (5.7) | 26.9 (5.3) | 27.3 (2.7) | (.0245*) |
| Median (IQR) | 26.8 (24-31) | 26.4 (23-30) | 25.9 (23-30) | 28.1 (26-29) | 7.9313 (.0189*) |
| Missing | 143 (17.0) | 129 (21.0) | 408 (15.8) | 5 (55.6) |  |
| Canadian Province |  |  |  |  | 28.144 (.3514**) |
| Alberta | 107 (12.7) | 86 (14.0) | 310 (12.0) | 1 (11.1) |  |
| British Columbia | 121 (14.4) | 71 (11.5) | 340 (13.2) | 2 (22.2) |  |
| Manitoba | 38 (4.5) | 35 (5.7) | 118 (4.6) | 0 (0.0) |  |
| Newfoundland and Labrador | 23 (2.7) | 16 (2.6) | 53 (2.1) | 0 (0.0) |  |
| New Brunswick | 18 (2.1) | 17 (2.8) | 69 (2.7) | 0 (0.0) |  |
| Nova Scotia | 35 (4.2) | 18 (2.9) | 75 (2.9) | 1 (11.1) |  |
| Northwest Territories | 0 (0.0) | 0 (0.0) | 1 (0.0) | 0 (0.0) |  |
| Nunavut | 0 (0.0) | 1 (0.2) | 0 (0.0) | 0 (0.0) |  |
| Ontario | 362 (42.9) | 257 (41.8) | 1162 (45.1) | 2 (22.2) |  |
| Prince Edward Island | 5 (0.6) | 5 (0.8) | 19 (0.7) | 0 (0.0) |  |
| Quebec | 89 (10.6) | 80 (13.0) | 320 (12.4) | 0 (0.0) |  |
| Saskatchewan | 34 (4.0) | 15 (2.4) | 75 (2.9) | 3 (33.3) |  |
| Yukon | 0 (0.0) | 1 (0.2) | 2 (0.1) | 0 (0.0) |  |
| Outside of Canada | 11 (1.3) | 13 (2.1) | 34 (1.3) | 0 (0.0) |  |
| Sex |  |  |  |  | 26.313 (<.0001**) |
| Male | 350 (41.5) | 337 (54.8) | 1239 (48.1) | 7 (77.8) |  |
| Female | 488 (57.9) | 275 (44.7) | 1327 (51.5) | 1 (11.1) |  |
| Other | 5 (0.6) | 3 (0.5) | 9 (0.3) | 1 (11.1) |  |
| Unknown | 0 (0.0) | 0 (0.0) | 3 (0.1) | 0 (0.0) |  |
| Highest Level of Education |  |  |  |  | 101.32 (<.0001*) |
| Elementary | 253 (30.0) | 154 (25.0) | 474 (18.4) | 1 (11.1) |  |
| Trade School | 65 (7.7) | 59 (9.6) | 161 (6.2) | 1 (11.1) |  |
| Community College | 223 (26.5) | 165 (26.8) | 616 (23.9) | 0 (0.0) |  |
| University(undergrad) | 211 (25.0) | 148 (24.1) | 905 (35.1) | 6 (66.7) |  |
| University (grad) | 89 (10.6) | 88 (14.3) | 421 (16.3) | 1 (11.1) |  |
| Unknown | 2 (0.2) | 1 (0.2) | 1 (0.0) | 0 (0.0) |  |
| Income Level |  |  |  |  | 47.439 (<.0001*) |
| < 30,000 | 166 (19.7) | 132 (21.5) | 393 (15.2) | 2 (22.2) |  |
| 30,000-50,000 | 185 (21.9) | 139 (22.6) | 476 (18.5) | 3 (33.3) |  |
| 51,000-80,000 | 217 (25.7) | 162 (26.3) | 647 (25.1) | 2 (22.2) |  |
| 81,000-120,000 | 152 (18.0) | 104 (16.9) | 584 (22.7) | 1 (11.1) |  |
| >120,000 | 120 (14.2) | 78 (12.7) | 473 (18.3) | 1 (11.1) |  |
| Unknown | 3 (0.4) | 0 (0.0) | 5 (0.2) | 0 (0.0) |  |
| Living Area |  |  |  |  | 7.6862 (.1038) |
| Rural | 194 (23.0) | 127 (20.7) | 487 (18.9) | 1 (11.1) |  |
| Suburban | 309 (36.7) | 236 (38.4) | 970 (37.6) | 3 (33.3) |  |
| Urban | 340 (40.3) | 252 (41.0) | 1121 (43.5) | 5 (55.6) |  |
| Do You Consider Yourself Healthy |  |  |  |  | 55.297 (<.0001*) |
| Not Sure | 137 (16.3) | 58 (9.4) | 234 (9.1) | 1 (11.1) |  |
| No | 143 (17.0) | 96 (15.6) | 317 (12.3) | 0 (0.0) |  |
| Yes | 563 (66.8) | 461 (75.0) | 2026 (78.6) | 8 (88.9) |  |
| Unknown | 0 (0.0) | 0 (0.0) | 1 (0.0) | 0 (0.0) |  |
| How many times per year do you go to the doctor? |  |  |  |  | 74866 (.2782) |
| 0 | 133 (15.8) | 94 (15.3) | 358 (13.9) | 1 (11.1) |  |
| 1 | 262 (31.1) | 226 (36.7) | 871 (33.8) | 3 (33.3) |  |
| 2-4. | 359 (42.6) | 236 (38.4) | 1083 (42.0) | 4 (44.4) |  |
| 5 or more | 89 (10.6) | 59 (9.6) | 266 (10.3) | 1 (11.1) |  |
| How frequently do you consider health benefits when you choose food to eat? |  |  |  |  | 126.83 (<.0001*) |
| Never | 57 (6.8) | 56 (9.1) | 67 (2.6) | 2 (22.2) |  |
| Sometimes | 437 (51.8) | 315 (51.2) | 1066 (41.3) | 3 (33.3) |  |
| Most of the time | 291 (34.5) | 196 (31.9) | 1160 (45.0) | 4 (44.4) |  |
| Always | 58 (6.9) | 48 (7.8) | 285 (11.1) | 0 (0.0) |  |
| What dietary pattern best describes the way you eat? |  |  |  |  | 30.221 (<.0001*) |
| Omnivorous | 755 (89.6) | 508 (82.6) | 2336 (90.6) | 7 (77.8) |  |
| Pescatarian | 44 (5.2) | 50 (8.1) | 111 (4.3) | 2 (22.2) |  |
| Vegetarian | 31 (3.7) | 41 (6.7) | 103 (4.0) | 0 (0.0) |  |
| Vegan | 9 (1.1) | 10 (1.6) | 22 (0.9) | 0 (0.0) |  |
| Unknown | 4 (0.5) | 6 (1.0) | 6 (0.2) | 0 (0.0) |  |
| **Indicates statistically significant differences between groups, ** Chi-squared approximation unreliable due to low frequencies.* | | | | | |

**Supplementary Table 9. Correlation Matrix statistics.**

| Variable 1 | Variable 2 | Correlation r | Exact P value | P value for matrix |
| --- | --- | --- | --- | --- |
| Number_Made | Number_Made | 1 | NA | NA |
| Number_Consumed | Number_Made | 0.09821892 | 3.85E-10 | < 0.0001 |
| Number_of_Children | Number_Made | 0.015548671 | 0.322832214 | NS |
| People_in_House | Number_Made | 0.020078954 | 0.201686363 | NS |
| Education | Number_Made | 0.101585229 | 9.44E-11 | < 0.0001 |
| Generations | Number_Made | -0.074398396 | 2.17E-06 | < 0.0001 |
| BMI | Number_Made | -0.030791636 | 0.05020458 | NS |
| Age | Number_Made | -0.056176367 | 0.000350827 | < 0.001 |
| Number_Made | Number_Consumed | 0.09821892 | 3.85E-10 | < 0.0001 |
| Number_Consumed | Number_Consumed | 1 | NA | NA |
| Number_of_Children | Number_Consumed | 0.139251531 | 0 | < 0.0001 |
| People_in_House | Number_Consumed | 0.071417894 | 5.45E-06 | < 0.0001 |
| Education | Number_Consumed | 0.160181878 | 0 | < 0.0001 |
| Generations | Number_Consumed | -0.151762284 | 0 | < 0.0001 |
| BMI | Number_Consumed | -0.061572706 | 8.91E-05 | < 0.0001 |
| Age | Number_Consumed | -0.240574925 | 0 | < 0.0001 |
| Number_Made | Number_of_Children | 0.015548671 | 0.322832214 | NS |
| Number_Consumed | Number_of_Children | 0.139251531 | 0 | < 0.0001 |
| Number_of_Children | Number_of_Children | 1 | NA | NA |
| People_in_House | Number_of_Children | 0.391920542 | 0 | < 0.0001 |
| Education | Number_of_Children | 0.124024565 | 2.44E-15 | < 0.0001 |
| Generations | Number_of_Children | -0.052843438 | 0.000773326 | < 0.001 |
| BMI | Number_of_Children | 0.001226234 | 0.937856065 | NS |
| Age | Number_of_Children | -0.182787173 | 0 | < 0.0001 |
| Number_Made | People_in_House | 0.020078954 | 0.201686363 | NS |
| Number_Consumed | People_in_House | 0.071417894 | 5.45E-06 | < 0.0001 |
| Number_of_Children | People_in_House | 0.391920542 | 0 | < 0.0001 |
| People_in_House | People_in_House | 1 | NA | NA |
| Education | People_in_House | 0.039547711 | 0.011887935 | < 0.05 |
| Generations | People_in_House | -0.052426095 | 0.000851218 | < 0.001 |
| BMI | People_in_House | -0.022068109 | 0.160534628 | NS |
| Age | People_in_House | -0.187239299 | 0 | < 0.0001 |
| Number_Made | Education | 0.101585229 | 9.44E-11 | < 0.0001 |
| Number_Consumed | Education | 0.160181878 | 0 | < 0.0001 |
| Number_of_Children | Education | 0.124024565 | 2.44E-15 | < 0.0001 |
| People_in_House | Education | 0.039547711 | 0.011887935 | < 0.05 |
| Education | Education | 1 | NA | NA |
| Generations | Education | -0.24667802 | 0 | < 0.0001 |
| BMI | Education | -0.094017564 | 2.09E-09 | < 0.0001 |
| Age | Education | -0.102412647 | 6.64E-11 | < 0.0001 |
| Number_Made | Generations | -0.074398396 | 2.17E-06 | < 0.0001 |
| Number_Consumed | Generations | -0.151762284 | 0 | < 0.0001 |
| Number_of_Children | Generations | -0.052843438 | 0.000773326 | < 0.001 |
| People_in_House | Generations | -0.052426095 | 0.000851218 | < 0.001 |
| Education | Generations | -0.24667802 | 0 | < 0.0001 |
| Generations | Generations | 1 | NA | NA |
| BMI | Generations | 0.134506106 | 0 | < 0.0001 |
| Age | Generations | 0.152000451 | 0 | < 0.0001 |
| Number_Made | BMI | -0.030791636 | 0.05020458 | NS |
| Number_Consumed | BMI | -0.061572706 | 8.91E-05 | < 0.0001 |
| Number_of_Children | BMI | 0.001226234 | 0.937856065 | NS |
| People_in_House | BMI | -0.022068109 | 0.160534628 | NS |
| Education | BMI | -0.094017564 | 2.09E-09 | < 0.0001 |
| Generations | BMI | 0.134506106 | 0 | < 0.0001 |
| BMI | BMI | 1 | NA | NA |
| Age | BMI | 0.135719012 | 0 | < 0.0001 |
| Number_Made | Age | -0.056176367 | 0.000350827 | < 0.001 |
| Number_Consumed | Age | -0.240574925 | 0 | < 0.0001 |
| Number_of_Children | Age | -0.182787173 | 0 | < 0.0001 |
| People_in_House | Age | -0.187239299 | 0 | < 0.0001 |
| Education | Age | -0.102412647 | 6.64E-11 | < 0.0001 |
| Generations | Age | 0.152000451 | 0 | < 0.0001 |
| BMI | Age | 0.135719012 | 0 | < 0.0001 |
| Age | Age | 1 | NA | NA |

**Supplementary Table 10.** Thematic categorization of open-ended responses on Q29 regarding perceived healthfulness of fermented foods. Responses were manually categorized and could be assigned to more than one theme when multiple concepts were expressed; therefore, category counts are not mutually exclusive and should not be summed to obtain the total number of respondents.

| **Theme** | **Description** | **Responses (N)** |
| --- | --- | --- |
| Gut health | References to gut or gastrointestinal health, digestion, bowel function, and related benefits | 488 |
| Microbes | References to probiotics, beneficial bacteria/microorganisms, microbial diversity, cultures, or microbial metabolites | 300 |
| Diet/nutrition | References to a healthy or balanced diet, nutritional value, vitamins, minerals, protein, fibre, or other nutrients | 125 |
| Hesitation/qualified benefit | Responses expressing uncertainty, moderation, variability among fermented foods, or qualification of the perceived benefit | 62 |
| Other | Other explanations for considering fermented foods beneficial that did not fall within the above themes | 446 |
| Reasons for not consuming fermented foods | Responses acknowledging potential benefits but describing personal reasons for avoiding fermented foods | 2 |
| Negative perception | Responses explicitly indicating that fermented foods were not considered healthy | 7 |
